# Cathepsin G and neutrophil elastase control *Leishmania infantum* parasitism through TLRs-dependent induction of IFN-α and TNF-α

**DOI:** 10.1038/s42003-026-10555-8

**Published:** 2026-06-29

**Authors:** Amy Goundry, Aislan C. Vivarini, Nathalia S. Rodrigues, Ulisses G. Lopes, Jeremy C. Mottram, Ana Paula C. A. Lima

**Affiliations:** 1 Laboratory of Biochemisty and Molecular Biology of Proteases, Instituto de Biofisica Carlos Chagas Filho, Universidade Federal do Rio de Janeiro, Rio de Janeiro, RJ Brazil; 2 Laboratory of Molecular Parasitology, Instituto de Biofisica Carlos Chagas Filho, Universidade Federal do Rio de Janeiro, Rio de Janeiro, RJ Brazil; 3 Department of Cellular and Molecular Biology, Instituto de Biologia, Universidade Federal Fluminense, Rio de Janeiro, RJ Brazil; 4 York Biomedical Research Institute and Department of Biology, University of York, York, UK

**Keywords:** Parasite host response, Mechanisms of disease, Infection

## Abstract

*Leishmania infantum* and *Leishmania donovani* are protozoan parasites that cause visceral leishmaniasis, a potentially fatal disease targeting the liver, spleen and bone marrow. To survive, these parasites manipulate host macrophage microbicidal responses and metabolic pathways. Here, we demonstrate that the host serine proteases neutrophil elastase (NE) and cathepsin G (CG), acting in concert with Toll-like receptors TLR2, TLR3, and TLR4, induce the inflammatory cytokines IFN-α and TNF-α to control *L. infantum* infection in macrophages. Conversely, in vivo experiments revealed that while NE and CG are necessary for inflammatory responses, including IL-12, nitric oxide, and CCL2 production, they unexpectedly promote splenic parasitism. CG-deficient mice failed to recruit Ly6C^hi^ myeloid cells to the spleen, providing a potential mechanism for their reduced parasite burden. Finally, our data suggest that unlike *L. donovani*, *L. infantum* cannot hijack cellular host serine peptidases and TLR signalling for survival, yet it remains dependent on these host enzymes to establish systemic spleen infection.

## Abstract (web summary)

Molecular insights into how serine proteases control *Leishmania infantum* parasitism in murine macrophages while promoting spleen infection.

## Introduction

The clinical outcome of infection with *Leishmania* spp. results from a combination of parasite and host factors, including the parasite species or strain, the host genetic background, and the immune response. In human visceral leishmaniasis (HVL), symptomatic individuals develop liver granulomas and significant alterations in the spleen architecture, leading to organ dysfunction that is potentially deadly. There are significant differences in the pathogenesis of HVL in India and East Africa, caused by *L. donovani*, and in Brazil, caused by *L. infantum*, which can also be observed in experimental murine models^1^. In HVL, severe bleeding and coagulopathy have been reported in Brazil, which enhances chance of death by 3.5-fold, greater than all other risk factors^2^. These conditions have been attributed to disseminated intravascular coagulation (DIC), and the severity of the disease in patients from Brazil has been associated with a higher concentration of serum inflammatory cytokines^2^. At the cellular level, mechanisms that enable or restrain *L. donovani* growth in macrophages were well explored, while less is known for *L. infantum*. Of relevance to this study, type I interferons (IFNs; IFN-α and IFN-β) have been reported as major regulators of macrophage parasitism by different *Leishmania* species. For example, while type I IFNs help to control experimental infection by *L. major*^3^, IFN-β was shown to favor infection by *L. amazonensis*^4^ and by *L. donovani*^5,6^, downstream of Toll-like (TLR) receptor stimulation. In experimental mice infections with *L. donovani*, type I IFNs were further identified as negative regulators of CD4^+^ T cells, and mice knockout for the common type I IFN receptor (*ifnar*^-/-^) have significantly lower parasite burdens^7^.

At the cellular level, we have previously uncovered that the host cell serine peptidase of the S1A family, neutrophil elastase (NE), is one of the upstream players in the pathway leading to IFN-β production in *Leishmania*-infected primary macrophages^5,6,8,9^. NE and cathepsin G (CG) are abundant not only in neutrophil granules but also in monocytes^10^, and perform proteolytic modification of cytokines and chemokines, receptor activation, and cleavage of coagulation factors^11^, which might be of relevance during leishmaniasis. We have previously shown that NE activity, TLR4, TLR2, and double-stranded RNA-activated protein kinase (PKR) take part in a molecular pathway that results in the production of IFN-β and in the expression of additional genes with relevance for macrophage parasitism^4,6,9^. The activation of the NE-TLR4 pathway, and the resulting IFN-β, has opposite consequences for parasite fate, depending on the *Leishmania* species. While NE and IFN-β are detrimental to *L. major*^8,9^, *L. donovani* requires NE activity and the resulting IFN-β for efficient growth in macrophages, and to develop high systemic parasite loads in mice^5,6^. In addition to NE-TLR4, TLR2 also contributes to the induction of IFN-β in macrophages infected with either *L. donovani*^6^ or *L. amazonensis*^4^, and is positively correlated with parasitism. Considering that the visceralizing species, *L. donovani* and *L. infantum*, can provoke varying pathologies in HVL, and might have evolved to trigger different molecular mechanisms at the cellular level to enable efficient parasitism, we were prompted to investigate which are the mechanistic requirements involving S1A peptidases for parasitism by *L. infantum* at the cellular level.

Using chemical inhibition and genetic approaches, we investigated how NE and CG affect the uptake, survival, and intracellular growth of *L. infantum* in macrophages and mapped the underlying molecular pathways associated with the infection in vitro. Furthermore, we assessed whether SPs affect the in vivo infection by using transgenic mice. We found that NE and CG, and TLRs 2, 3, and 4, act in conjunction in a molecular pathway induced in macrophages upon *L. infantum* infection, resulting in the production of TNFα and IFNα, which limits *L. infantum* development at the cellular level, while relying of these SPs to establish splenic infection in mice.

## Results

**Fig. 1:**
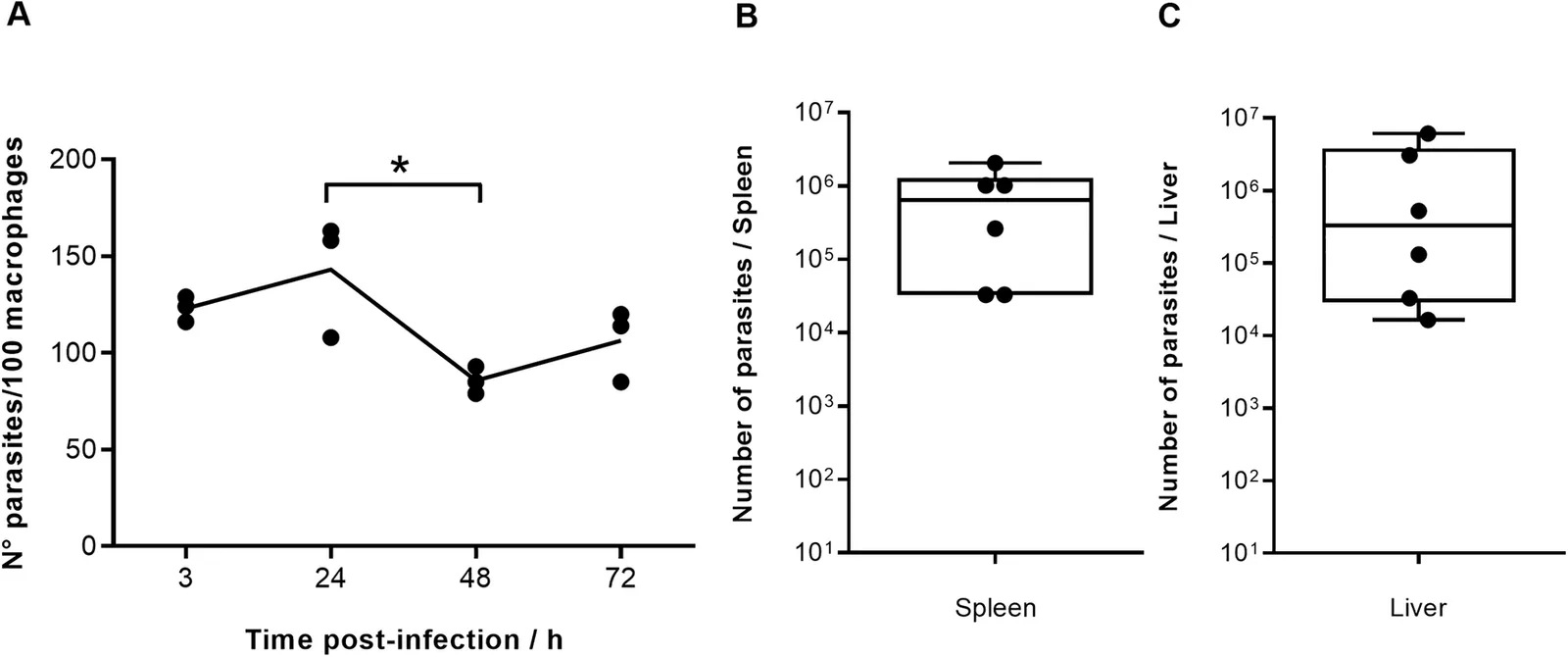
*L. infantum* PP75 strain is infective to macrophages and mice of the C57BL/6 model. **A** Peritoneal thioglycolate-recruited macrophages from C57BL/6 mice cultivated on glass coverslips overnight were infected with promastigotes of *L. infantum* at a 5:1 parasite:macrophage ratio in RPMI-BSA at 37°C. After 3 h, the monolayers were washed to remove extracellular parasites, fixed with methanol and Giemsa-stained, or further incubated for the indicated times in RPMI-FCS at 37°C before fixation and staining. The number of intracellular parasites was determined under the light microscope. The experiment was repeated 4 independent times, and the graph shows one experiment with three biological replicates (means ± SD). The 4 independent experiments are shown in Supplementary Fig 1. Statistical significance was assessed using one-way ANOVA with the Bonferroni multiple comparisons test. Significant differences between timepoints are indicated; *P < 0.05. **B, C** C57BL/6 mice (*n* = 6) were infected by retro-orbital inoculation of *L. infantum* promastigotes (10^7^ parasites/animal in PBS). After 1 week, the spleens and livers were removed, homogenized, and parasite loads were assessed by limiting dilution and represented as parasites/organ. Graphs are from two independent infections (*n* = 3 animals/group). Each point represents an individual mouse, and graphs are shown as box and whisker plots with the median (center line), lower and upper quartiles (25th and 75th percentiles; box limits), and min/max (whiskers).

**Fig. 2:**
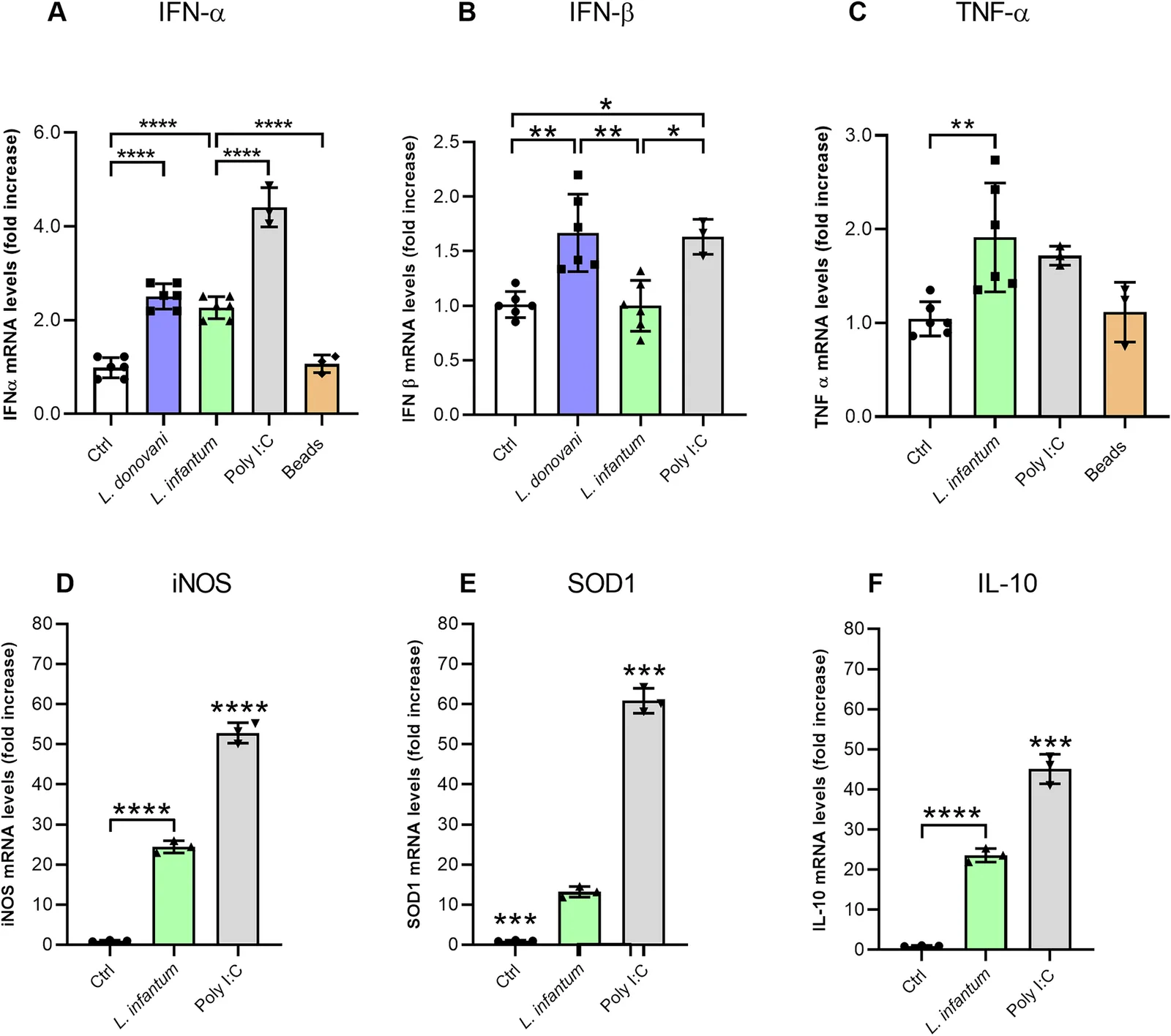
*L. infantum* and *L. donovani* induce opposite cytokine profiles from macrophages. Peritoneal thioglycolate-recruited macrophages from C57BL/6 mice cultured overnight were infected with promastigotes of *L. donovani* and *L. infantum* at a 10:1 parasite:macrophage ratio in RPMI-BSA at 37°C for 2 h. Cultures were then washed to remove extracellular parasites and incubated in RPMI-BSA for a further 4 h, after which total RNA was extracted. Where indicated, carboxylated microsphere beads (0.2 μm) were added at 1:2000 dilution to the macrophages. cDNA was made and used as templates in qPCR for the determination of the relative mRNA levels for **A** IFN-α, **B** IFN-β, **C** TNF-α, **D** iNOS, **E** SOD1, and **F** IL-10. Uninfected cells were used as control (Ctrl) levels, and cells pretreated with poly I:C (25 µg mL^-1^) were used as a positive control. The experiment was repeated 3 times (2 biological replicates each). Biological replicates were compiled and means ± SD are shown. **A**–**C** *n* = 6, **D**–**F** *n* = 3. Statistical significance was assessed using one-way ANOVA with Bonferroni multiple comparisons test (alpha level of 0.05); test *P*-values are **A** P = 0.0069; **B** P = 0.0002; **C** *P* = 0.0001; **D** *P* < 0.0001; **E** *P* < 0.0001; and **F** *P* < 0.0001. Significant differences between groups are shown over lines, while asterisks over bars indicate significant differences compared to all other groups; *P < 0.05, **P < 0.01, ***P < 0.001.

We reported that *L. donovani*-induced IFN-β expression in macrophages is crucial for parasite growth/development in the infected cell, and this is conveyed upstream through the activation of a pathway that requires NE activity and TLR4^5^. In contrast, the activation of the NE-TLR4 pathway by *L. major*, leading to IFN-β and TNF-α expression, results in parasite killing^8^. To investigate how this molecular mechanism applies to macrophage infection by *L. infantum*, and to dissect the underlying pathways, we first sought to compare the pattern of expression of inflammation-related genes by peritoneal macrophages from C57BL/6 mice infected in vitro with two visceralizing *Leishmania* species. For this, we used an *L. infantum* reference strain from Brazil and a Sudanese *L. donovani* strain, the latter of which had been the focus of previous studies from our group^5,6^. We first verified that the *L. infantum* strain was able to infect primary macrophages obtained from C57BL/6 mice and cause in vivo infection in the same mouse strain (Fig. 1 and Supplementary Fig 1). Next, we performed parallel macrophage infections with the two species to determine the pattern of expression of type 1 IFNs and of additional inflammatory genes. To enable comparisons to be made with data of our previous studies with *L. donovani*^5,6^, we opted to determine gene expression at six hours after parasite uptake. We promptly detected mRNA for type I interferons, with IFN-α induced at similar levels from macrophages infected with either species (Fig. 2A), while IFN-β was induced exclusively by *L. donovani* (Fig. 2B). Higher TNF-α mRNA (Fig. 2C) was detected in *L. infantum*-infected macrophages than in uninfected controls. Exposure of macrophages to inert beads had no effect in the expression of those cytokines, indicating that the induction of mRNA was a specific response to infection. The expression of inducible nitric oxide synthase (iNOS) (Fig. 2D) was significantly induced in macrophages infected with *L. infantum*, as compared to uninfected controls. To check if counter-inflammatory responses were being triggered, we evaluated superoxide dismutase (SOD1) (Fig. 2E), related to the mechanisms of immune evasion by *L. amazonensis*^12^, and IL-10 (Fig. 2F), associated with the control of *L. donovani* parasitism and pathology^13^. *L. infantum* also induced SOD1 and IL-10 in infected macrophages. These observations indicated that macrophages infected with *L. infantum* seem prone to an inflammatory response (i.e., higher levels of TNF-α, IFN-α, and iNOS), while not producing IFN-β, which is opposite for that observed in *L. donovani* infection.

**Fig. 3:**
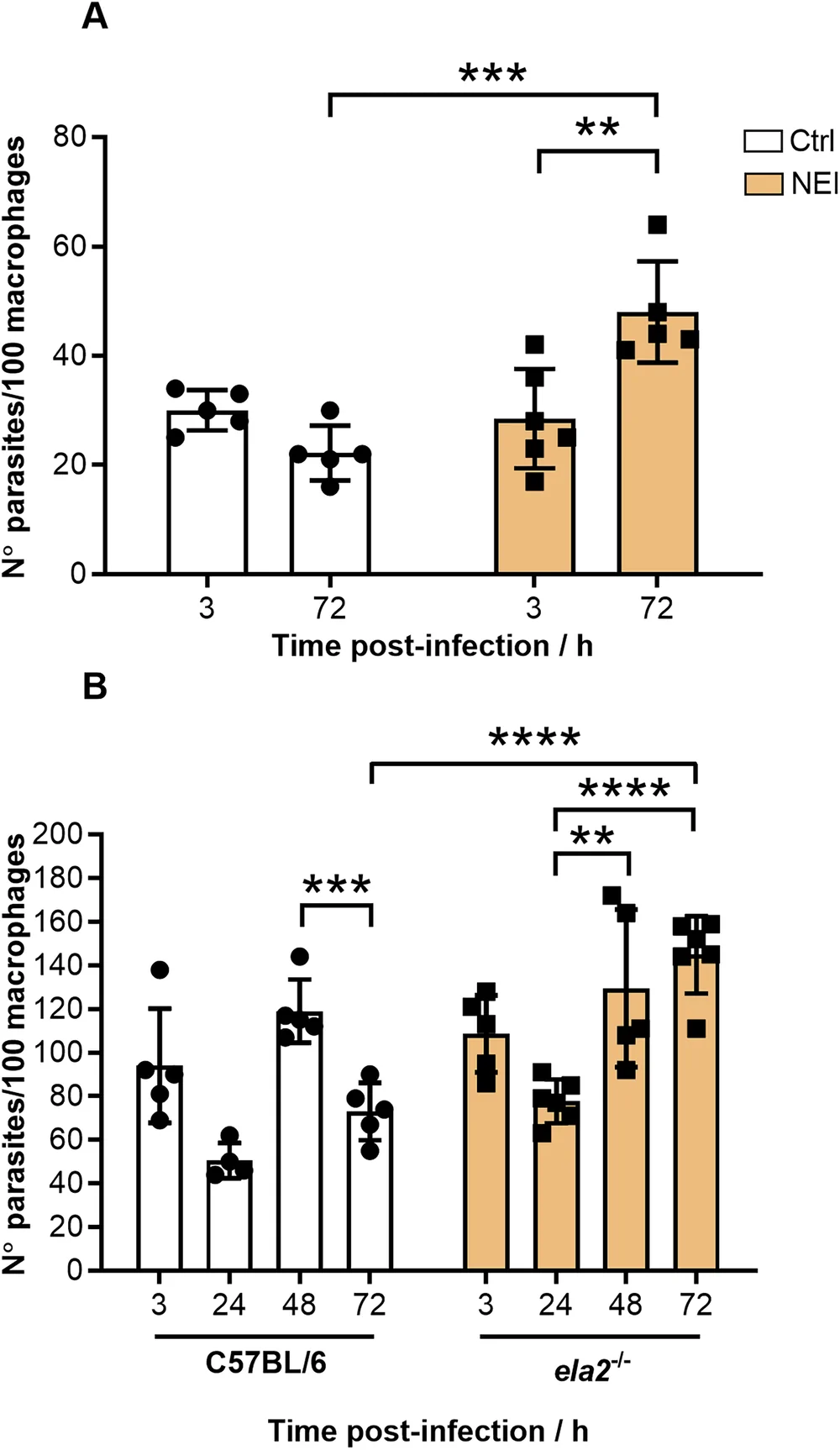
Neutrophil elastase contributes to the control of *L. infantum* long-term infection in macrophages. Peritoneal thioglycolate-recruited macrophages from **A**, **B** C57BL/6 mice and **B** from neutrophil elastase (NE)-knockout mice (*ela2*^-/-^) cultivated on glass coverslips overnight were infected with promastigotes of *L. infantum* at a 5:1 parasite:macrophage ratio in RPMI-BSA at 37°C. After 3 h, the monolayers were washed to remove extracellular parasites, fixed with methanol and Giemsa-stained, or further incubated for the indicated times in RPMI-FCS at 37°C before fixation and staining. The number of intracellular parasites was determined under the light microscope. In (**A**) the irreversible inhibitor of NE, MeOSuc-AlaAlaProVal-CMK (NEI; 10 µM; Calbiochem) was added 5 min prior to infection of macrophages from C57BL/6 mice. Each experiment was performed twice (3 biological replicates and 2 biological replicates). The graphs show biological replicates compiled (means ± SD). Statistical significance was assessed using two-way ANOVA with Tukey’s multiple comparisons test. Significant differences between groups at the same timepoint and timepoints within the same group are indicated; **P < 0.01, ***P < 0.001, ****P < 0.0001.

Considering that there is a correlation between NE and IFN-β for sustained macrophage infection by *L. donovani*^5,6^ and that we observed opposite macrophage response in infections with both species, we asked whether NE plays a role in *L. infantum* infection (Fig. 3). Infection in macrophages from C57BL/6 mice showed that parasites did not grow within 72 h after parasite uptake (Fig. 3A). However, a much higher parasite burden at 72 hours occurred in the presence of an irreversible chloro-methyl ketone peptidyl inhibitor of NE, MeOSuc-AAPV-CMK, suggesting that enzyme inhibition favored parasite survival and/or proliferation. This was further addressed by analyzing the timecourse of infection using macrophages derived from NE-knockout mice (*ela2*^-/-^), in comparison to those from the background C57BL/6 mice (Fig. 3B). There was no difference in the uptake of parasites by the macrophages from the two mouse lines after 3 hours, and a proportion of the intracellular parasites were killed by 24 h, presumably of the internalized promastigote forms. At 48 hours, an increase in the number of intracellular parasites occurred, denoting amastigote proliferation. However, while a 25% reduction in the parasite burden was observed in macrophages from C57BL/6 mice at 72 h, the burden remained unchanged in macrophages from *ela2*^-/-^ mice, confirming that NE contributes to the control of *L. infantum* inside macrophages. Although the levels of parasite internalization differed between assays, the pattern of infection within the macrophages over 72 h was reproducible. Such variation in the baseline phagocytosis of parasites is expected, since the activation levels of primary macrophages recruited with thioglycolate might differ between experiments. Independent experiments showing the kinetics of parasite infection/survival in macrophages of C57BL/6 mice are depicted in Supplementary Fig. 1.

**Fig. 4:**
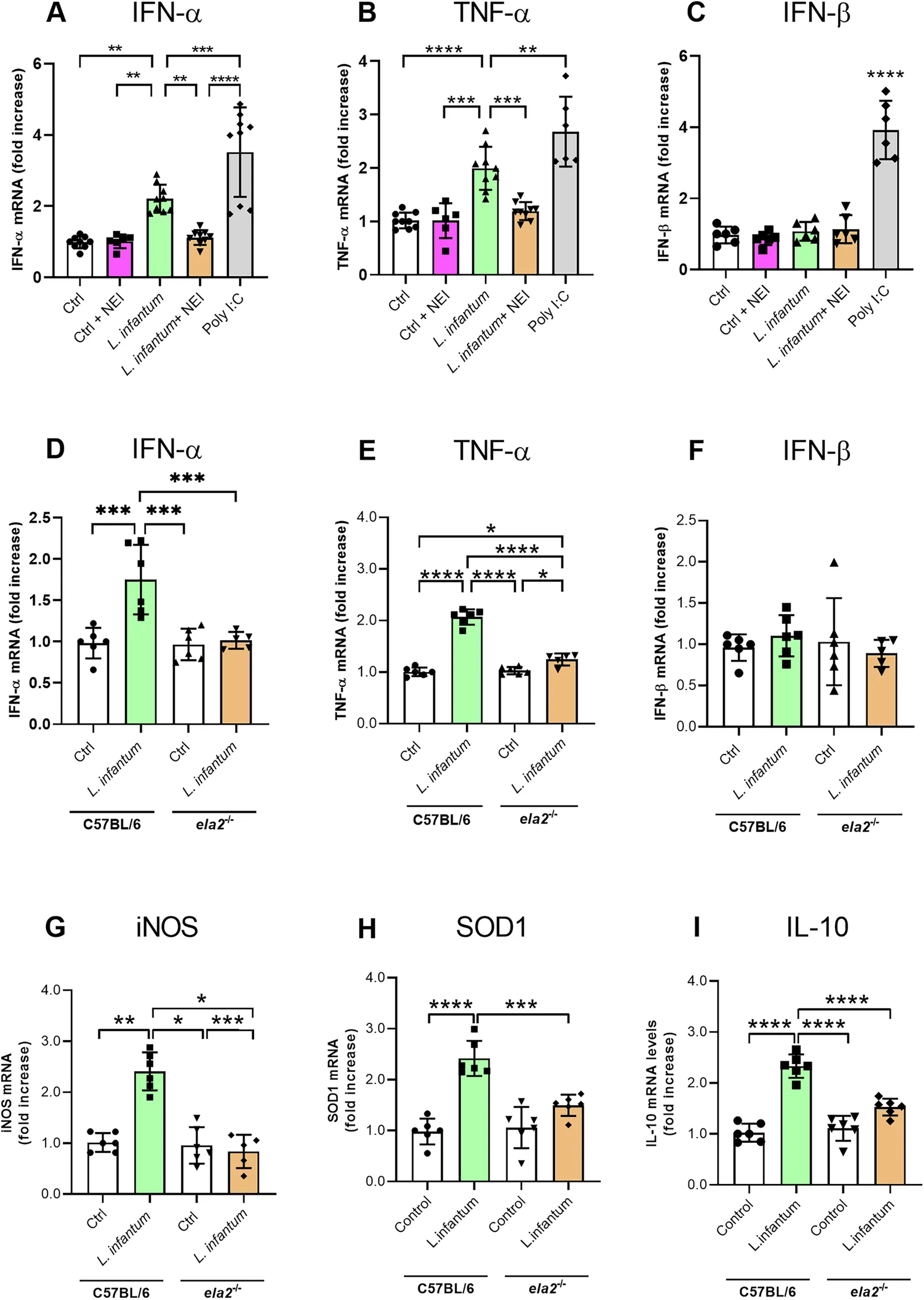
Neutrophil elastase activity is required for the induction of pro-inflammatory cytokines in *L. infantum*-infected macrophages. Peritoneal thioglycolate-recruited macrophages cultured overnight were infected with promastigotes of *L. infantum* at a 10:1 parasite:macrophage ratio in RPMI-BSA at 37°C for 2 h. Cultures were then washed to remove extracellular parasites and incubated in RPMI-BSA for a further 4 h, after which total RNA was extracted. cDNA was made and used as templates in qPCR for the determination of the relative mRNA levels for **A**, **D** IFN-α, (**B**, **E**) TNF-α, (**C**, **F**) IFN-β, (**G**) iNOS, (**H**) SOD1 and (**I**) IL-10. In (**A**–**C**), macrophages were from C57BL/6 mice and where indicated, cells were pretreated for 5 min with the irreversible inhibitor of neutrophil elastase, MeOSuc-AlaAlaProVal-CMK (NEI; 10 µM; Calbiochem) prior to the addition of parasites. In (**D**–**I**), macrophages were obtained from C57BL/6 and NE-knockout mice (*ela2*^-/-^). Uninfected cells were used as control (Ctrl) levels, and cells pretreated with poly I:C (25 µg mL^−^^1^) were used as positive control. The experiment was performed 3 times, each time with 2 or 3 biological replicates. The graphs show the means ± SD of compiled biological replicates (**A**, **B** *n* = 9 samples, **C**–**I**, *n* = 6 samples). Statistical significance was assessed using one-way ANOVA followed by Bonferroni multiple comparisons test (alpha level of 0.05); **A**–**C**, **E**, **G**, **I**, and **J** *P* < 0.0001; **D** *P* = 0.0101; **F** *P* = 0.3144; and **H** *P* = 0.0001. Significant differences between groups are shown over lines, while asterisks over bars indicate significant differences compared to all other groups; *P < 0.05, **P < 0.01, ***P < 0.001, ****P < 0.0001.

To address if NE took part in the induction of pro-inflammatory cytokines upon *L. infantum* infection, we determined the mRNA levels for type I IFNs and TNF-α (Fig. 4). We found that NE contributes to the induction of inflammatory IFN-α (Fig. 4A, D) and TNF-α (Fig. 4B, E) by *L. infantum*, which might contribute to parasite killing and/or prevention of growth observed at 72 h. Again, we did not detect induction of IFN-β by *L. infantum*-infected macrophages (Fig. 4C, F). While induced in macrophages from C57BL/6 mice, iNOS, SOD1, and IL-10 were not induced in *ela2*^-/-^ macrophages upon infection (Fig. 4G–I). Even though fold-increase of iNOS mRNA in infected macrophages in relation to uninfected controls were lower than in Fig. 2, the observation that the parasite induces iNOS in C57BL/6 macrophages was consistent in both experiments.

**Fig. 5:**
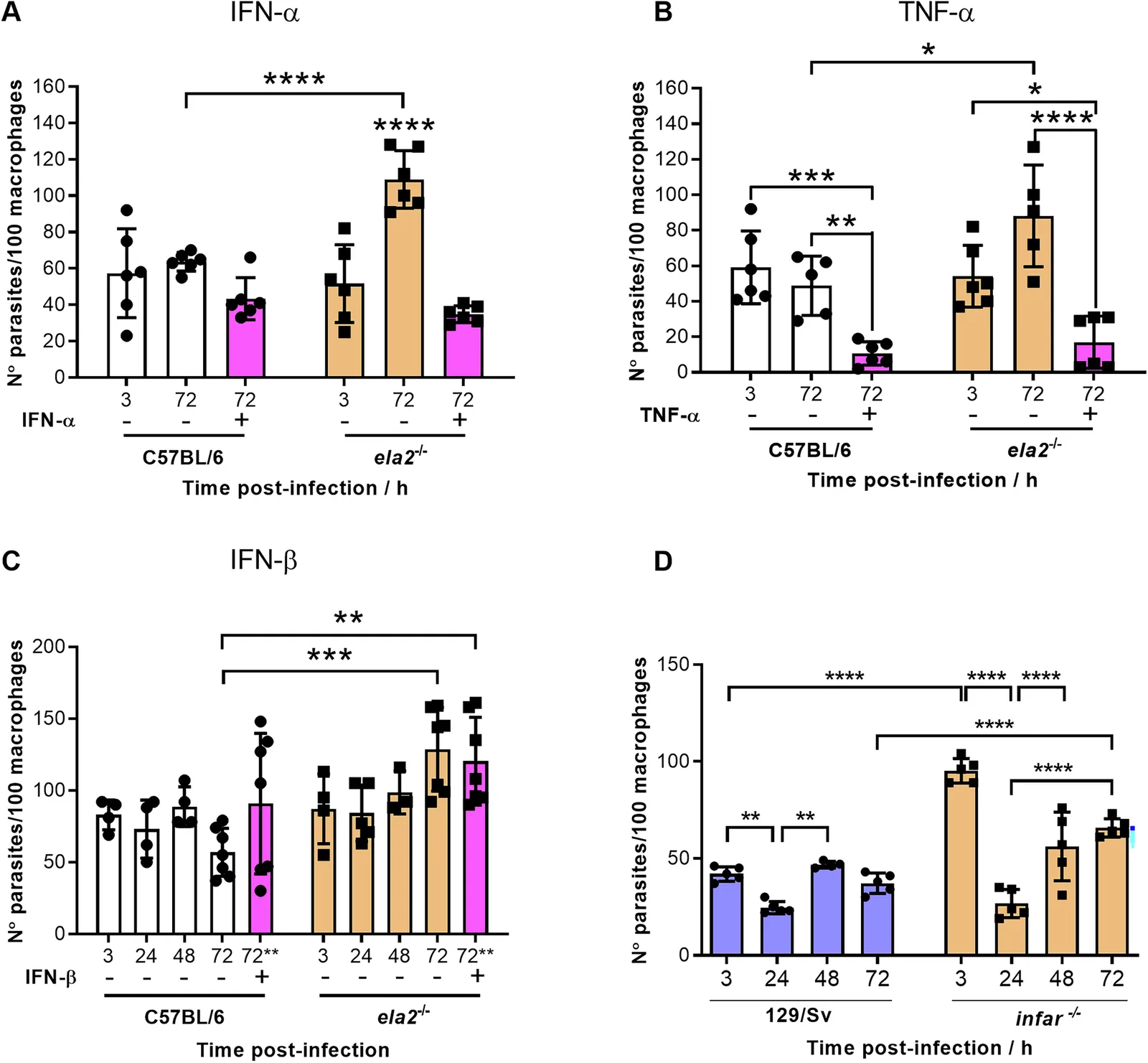
IFN-α and TNF-α kill *L. infantum* in infected macrophages. Peritoneal thioglycolate-recruited macrophages from **A**–**C** C57BL/6 and neutrophil elastase-knockout mice (*ela2*^-/-^), or **D** 129/Sv and type I interferon receptor (IFNAR)-knockout mice (*ifnar*^-/-^) cultivated on glass coverslips overnight, were infected with promastigotes of *L. infantum* at a 5:1 parasite: macrophage ratio in RPMI-BSA at 37 °C. After 3 h, the monolayers were washed to remove extracellular parasites, fixed with methanol and Giemsa-stained, or further incubated for the indicated times in RPMI-FCS at 37°C before fixation and staining. The number of intracellular parasites was determined under the light microscope. Where indicated, **A** IFN-α (200 ng mL^−^^1^; eBioscience), **B** TNF-α (200 ng mL^−^^1^; BioLegend), or **C** IFN-β (200 ng mL^−1^; BioLegend) were added to cultures after they had been infected for 3 h and washed for removal of extracellular parasites. Experiments were performed twice (3 biological replicates each) (**A, B**), or **C** three times (2 or 3 biological replicates each). The graphs show the means ± SD of biological replicates compiled. **A**, **B** Data represent paired samples from *n* = 6 biologically independent samples analyzed in two independent experiments. *P < 0.05, ****P < 0.0001. Statistical significance was assessed using Two-way ANOVA followed by Tukey’s multiple comparisons test for comparisons between time points within each group, and Sidak’s multiple comparisons test for comparisons between groups at each time point. **C** Data represent *n* = 4 or *n* = 8 biologically independent samples obtained from three independent experiments. **P < 0.01, ***P < 0.001 (Two- way ANOVA followed by Tukey’s multiple comparisons test). **D** Data represent samples from *n* = 5 biologically independent samples. *P < 0.05, **P < 0.01, ***P < 0.001, ****P < 0.0001.

The assumption that the pro-inflammatory cytokines induced in the macrophages from C57BL/6 mice affected the intracellular survival of *L. infantum* was directly tested by the addition of exogenous cytokines immediately after parasite uptake (Fig. 5). *L. infantum* was sensitive to IFN-α, whose induction depends on NE, as exogenous IFN-α reverted the sustained growth in *ela2*^-/-^ macrophages (Fig. 5A). A similar pattern was observed for TNF-α (Fig. 5B), which provoked a drastic reduction in the number of intracellular parasites at 72 h in both C57BL/6 and *ela2*^-/-^ macrophages. In contrast, there was no effect on parasite burdens upon addition of IFN-β (Fig. 5C), supporting the notion that, while this type I IFN is crucial for infection by *L. donovani* and *L. amazonensis*, it is dispensable for the infection by *L. infantum*, at least so in the levels tested here. A time-course infection of macrophages from mice deficient in the IFN-α/β receptor alpha chain (*ifnar*^-/-^), one of the two chains of the receptor for type I IFNs, further showed that at the 72 h timepoint, type I IFN signaling, presumably through IFN-α, is required to control *L. infantum* burden in macrophages (Fig. 5D).

**Fig. 6:**
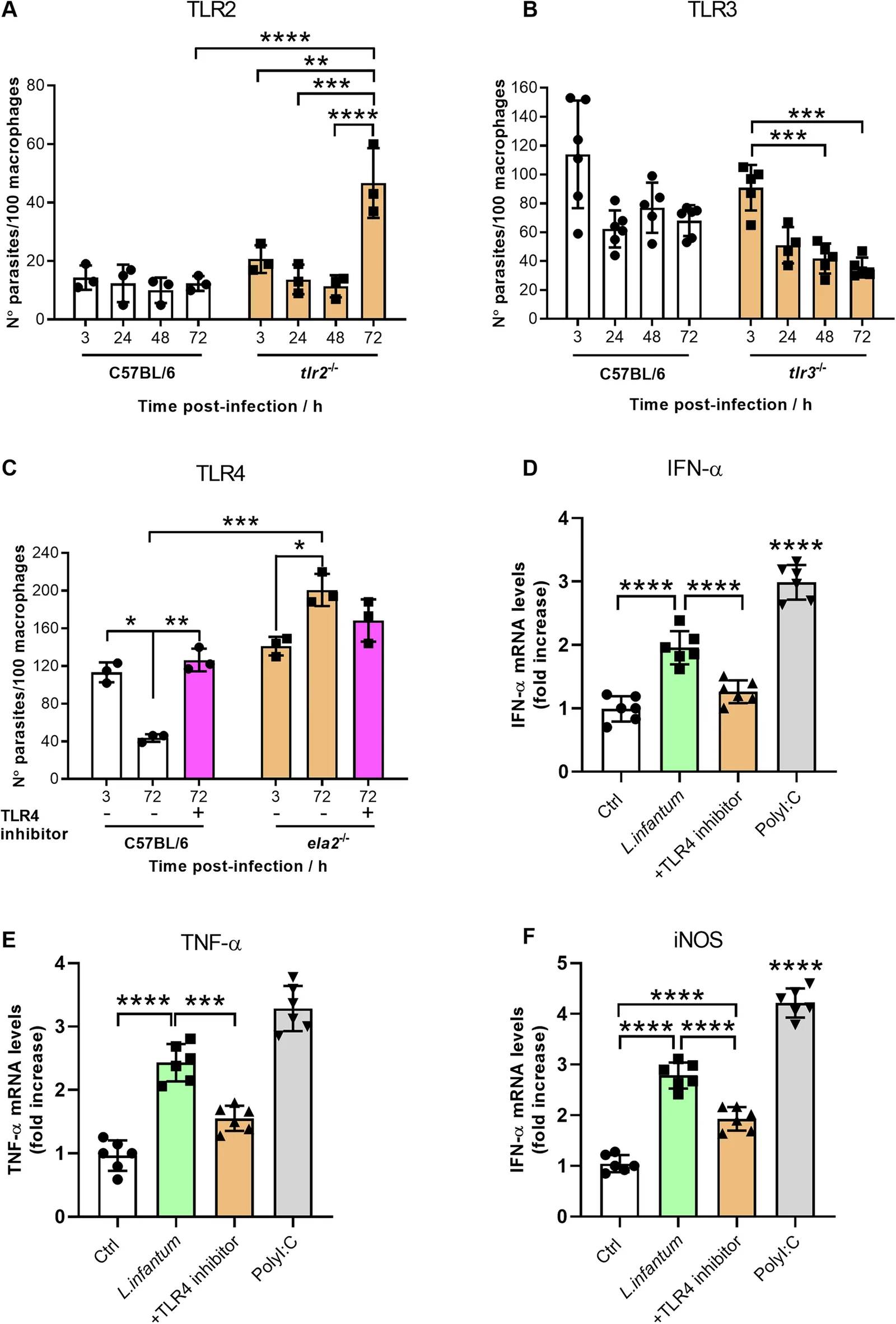
TLR2 and TLR4 control *L. infantum* infection in macrophages, while TLR3 contributes to infection. Peritoneal thioglycolate-recruited macrophages from C57BL/6 mice and from **A** Toll-like receptor 2 (TLR2)-knockout (*tlr2*^-/-^), **B** TLR3-knockout (*tlr3*^-/-^), or **C**, **D** neutrophil elastase (NE)-knockout mice (*ela2*^-/-^) were cultivated on glass coverslips overnight and then infected with promastigotes of *L. infantum* at a 5:1 parasite: macrophage ratio in RPMI-BSA at 37 °C. After 3 h, the monolayers were washed to remove extracellular parasites, fixed with methanol, and Giemsa-stained, or further incubated for the indicated times in RPMI-FCS at 37°C before fixation and staining. The number of intracellular parasites was determined under the light microscope. Where indicated, macrophages were **C**–**F** pretreated with the inhibitor of TLR4, Restorvid (TAK-242; 1 nM; eBioscience) for 15 min prior to infection. Experiments were performed twice, each with 3 biological replicates. **B** The graph shows data compiled for biological replicates (means ± SD). In (**A**), (**C**), the graphs show 3 biological replicates. The independent experiments are shown in Supplementary Fig. 2. **D**–**F** Experiments were performed three times, each with 2 biological replicates; the graphs show compiled biological replicates (means ± SD). Statistical significance was assessed using two-way ANOVA followed by Sidak’s multiple comparisons test. Significant differences between groups at the same timepoint/under the same treatment and between timepoints/treatments within the same group are indicated; *P < 0.05, **P < 0.01, ***P < 0.001, ****P < 0.0001.

**Fig. 7:**
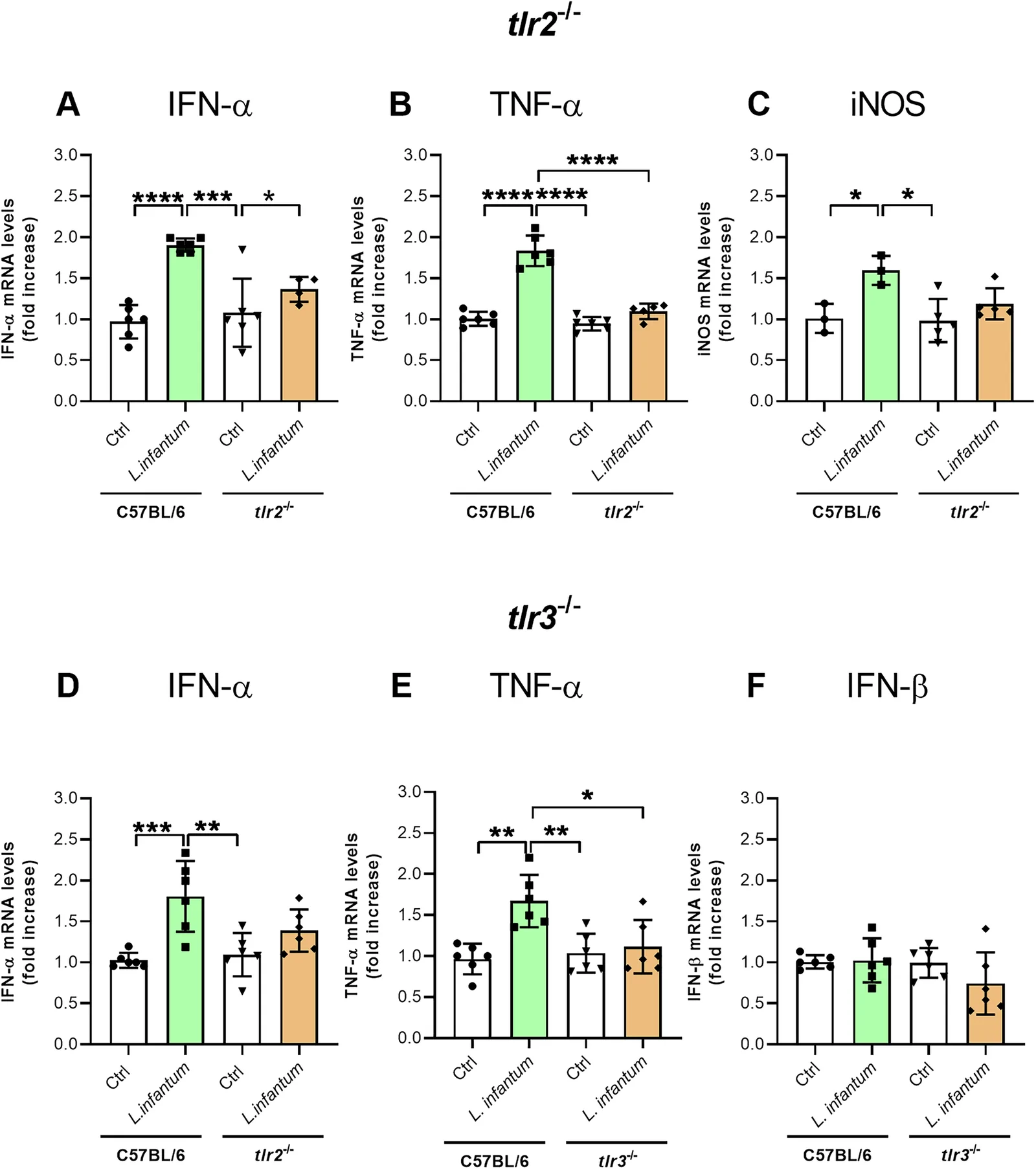
The Toll-like receptors are required for the induction of pro-inflammatory cytokines in *L. infantum*-infected macrophages. Thioglycolate-recruited macrophages from C57BL/6 mice and from **A**–**C** Toll-like receptor 2 (TLR2)-knockout (*tlr2*^-/-^) or **D**–**F** TLR3-knockout (*tlr3*^-/-^) mice were infected with promastigotes of *L. infantum* at a 10:1 parasite:macrophage ratio in RPMI-BSA at 37 °C for 2 h. Cultures were then washed and incubated in RPMI-BSA for a further 4 h, after which total RNA was extracted. cDNA was made and used as templates in qPCR for the determination of the relative mRNA levels for **A**, **D** IFN-α, **B**, **E** TNF-α, **C** iNOS, and **F** IFN-β. Uninfected cells were used as control (Ctrl) levels. Experiments were performed three times, each with 2 biological replicates. Data represent *n* = 6 biologically independent replicates compiled from three independent experiments. The graphs show biological replicates compiled (means ± SD). Statistical significance was assessed using one-way ANOVA with Bonferroni multiple comparisons test (alpha level of 0.05); **A**, **B**, **D** *P* < 0.0001; **C** *P* = 0.0013; **E** *P* = 0.0060; **F** P = 0.0084; and **G** *P* = 0.7400. Significant differences were shown between groups over lines; *P < 0.05, **P < 0.01, ***P < 0.001, ****P < 0.0001.

Since the molecular components that were previously shown to be beneficial for *L. donovani* infection of macrophages^5,6^ did not seem to be beneficial for the *L. infantum*-macrophage infection, we asked how this relates to the requirement of different TLRs for infection. In agreement with a mechanistic link between TLR2 and NE, macrophages derived from TLR2-knockout mice (*tlr2*^*-/-*^) displayed a significant increase in the parasite burden after 72 h, as compared to macrophages from background C57BL/6 mice (Fig. 6A and Supplementary Fig. 2). In contrast, parasites were unable to grow in macrophages from TLR3-knockout mice (*tlr3*^*-/-*^), indicating that this receptor contributes to infection (Fig. 6B). The role of TLR4 was verified upon addition of a selective inhibitor of TLR4 signaling, Resatorvid^14^, which increased parasite burdens by 100%, as compared to untreated macrophages (Fig. 6C and Supplementary Fig. 2). Although NE is primarily an enzyme present in neutrophil granules, it is knowingly present in mononuclear cells^10^, and we have previously detected it at the surface of macrophages, colocalized with TLR4 and *L. donovani*, enabling the NE-TLR4 platform to be triggered upon parasite infection^5^. Next, we sought to address the role of TLR4 in the induction of the expression of the leishmanicidal cytokines described in the above results. We observed that inhibition of TLR4 signaling with resatorvid prevented the induction of IFN-α (Fig. 6D), TNF-α (Fig. 6E) and iNOS (Fig. 6F). These results show that both NE and TLR4 are required for the induction of IFN-α and TNF-α, and to control parasite growth in macrophages. The findings that the absence of NE (Fig. 3) or the blockade of TLR4 function alone (Fig. 6C), and affect the NE-TLR4 axis, allowing *L. infantum* to reach higher numbers in macrophages, support the notion that NE and TLR4 act in conjunction to promote the anti-parasitic effect. We next analyzed if/how additional TLRs contributed to parasite-induced gene expression. As seen in the *ela2*^-/-^ macrophages, the expression of IFN-α, TNF-α, and iNOS was not induced in the *tlr2*^-/-^ macrophages upon infection as compared to the C57BL/6 macrophages (Fig. 7A–C). A similar pattern was observed for the contribution of TLR3. IFN-α and TNF-α did not seem to be induced in *tlr3*^-/-^ macrophages, while significantly induced in infected C57BL/6 macrophages (Fig. 7D, E). Again, IFN-β mRNA was not detected upon infection and this remained unchanged in the absence of TLR3 (Fig. 7F), indicating that this TLR also plays a role in the induction of those genes by *L. infantum*.

**Fig. 8:**
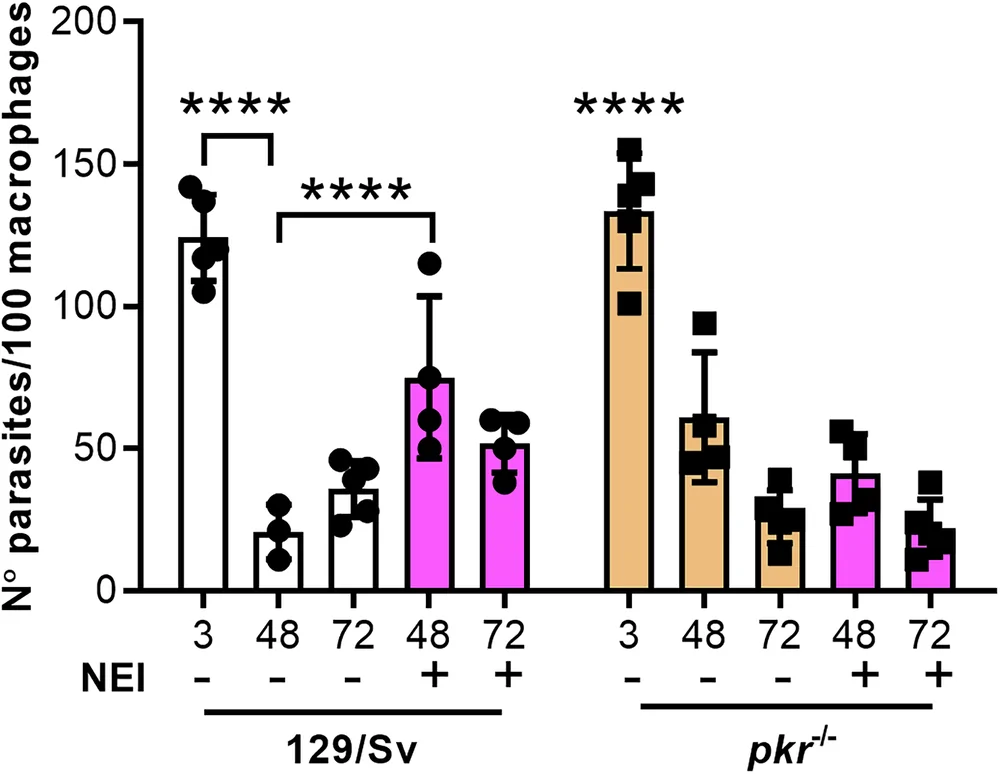
The leishmanicidal effect of neutrophil elastase is conveyed through protein kinase R. Peritoneal thioglycolate-recruited macrophages from 129/Sv and protein kinase R (PKR)-knockout mice (*pkr*^-/-^) cultivated on glass coverslips were infected with promastigotes at a 5:1 parasite: macrophage ratio in RPMI-BSA at 37 °C. After 3 h, the monolayers were washed to remove extracellular parasites, fixed with methanol and Giemsa-stained, or further incubated for the indicated times in RPMI-FCS at 37°C before fixation and staining. The number of intracellular parasites was determined under the light microscope. Where indicated, the irreversible inhibitor of neutrophil elastase, MeOSuc-AlaAlaProVal-CMK (NEI; 10 µM; Calbiochem), was added 5 min prior to infection. Data represent *n* = 5 biologically independent samples obtained from three independent experiments. The experiments were performed three times, with 3 or 2 biological replicates. The graphs show biological replicates of 2 experiments compiled (means ± SD). One experiment is shown in Supplemental Fig. 4. Statistical significance was assessed using two-way ANOVA followed by Sidak’s and Tukey’s (between the timepoints for each group) and Sidak’s (between the groups at each timepoint) multiple comparisons tests. Asterisks over bars indicate significant differences compared to the data for all other timepoints or treatments within the group; *P < 0.05, **P < 0.01, ***P < 0.001, ****P < 0.0001.

The cytoplasmic kinase PKR, associated with responses to double-stranded RNA viruses, was identified as the main component of the signaling pathway leading to type I IFNs in macrophage infection by *L. amazonensis*^15^. We showed that the NE-TLR4 pathway is also conveyed through PKR, and the resulting pattern of gene expression varies according to the *Leishmania* species, i.e., in a model of transgenic *L. major* it leads to IFN-β, TNF-α, and parasite killing^9^, while in *L. donovani* infections it results in IFN-β, IL-10, SOD1, OASL2, and parasite growth^6^. We next verified if PKR was associated with the control of *L. infantum* in macrophages. At 48 h, the parasite burden in macrophages of PKR-knockout mice (*pkr*^-/-^) was higher than that of macrophages of the background 129/Sv mice (Fig. 8 and Supplementary Fig. 3), linking this kinase to the anti-parasite activity. At 72 h, burdens were not sustained in 129/Sv macrophages, which agrees with a high leishmanicidal activity for macrophages from this genetic background. We tested whether NE activity played a role in the leishmanicidal phenotype of those macrophages by adding the NE inhibitor. This treatment increased parasite burdens in 129/Sv macrophages at 48 h, which remained elevated at 72 h, as compared to untreated controls, supporting a role for NE in controlling parasitism. In *pkr*^-/-^ macrophages, we found higher parasitism at 48 h as compared to 129/Sv macrophages, confirming that PKR contributes to the responses leading to parasite control. In contrast, the NE inhibitor did not further enhance parasite burden at 48 h in those macrophages, supporting the notion that the leishmanicidal effect of NE is conveyed through PKR, and in its absence, enzyme inhibition does not provide further advantage to sustain infection.

**Fig. 9:**
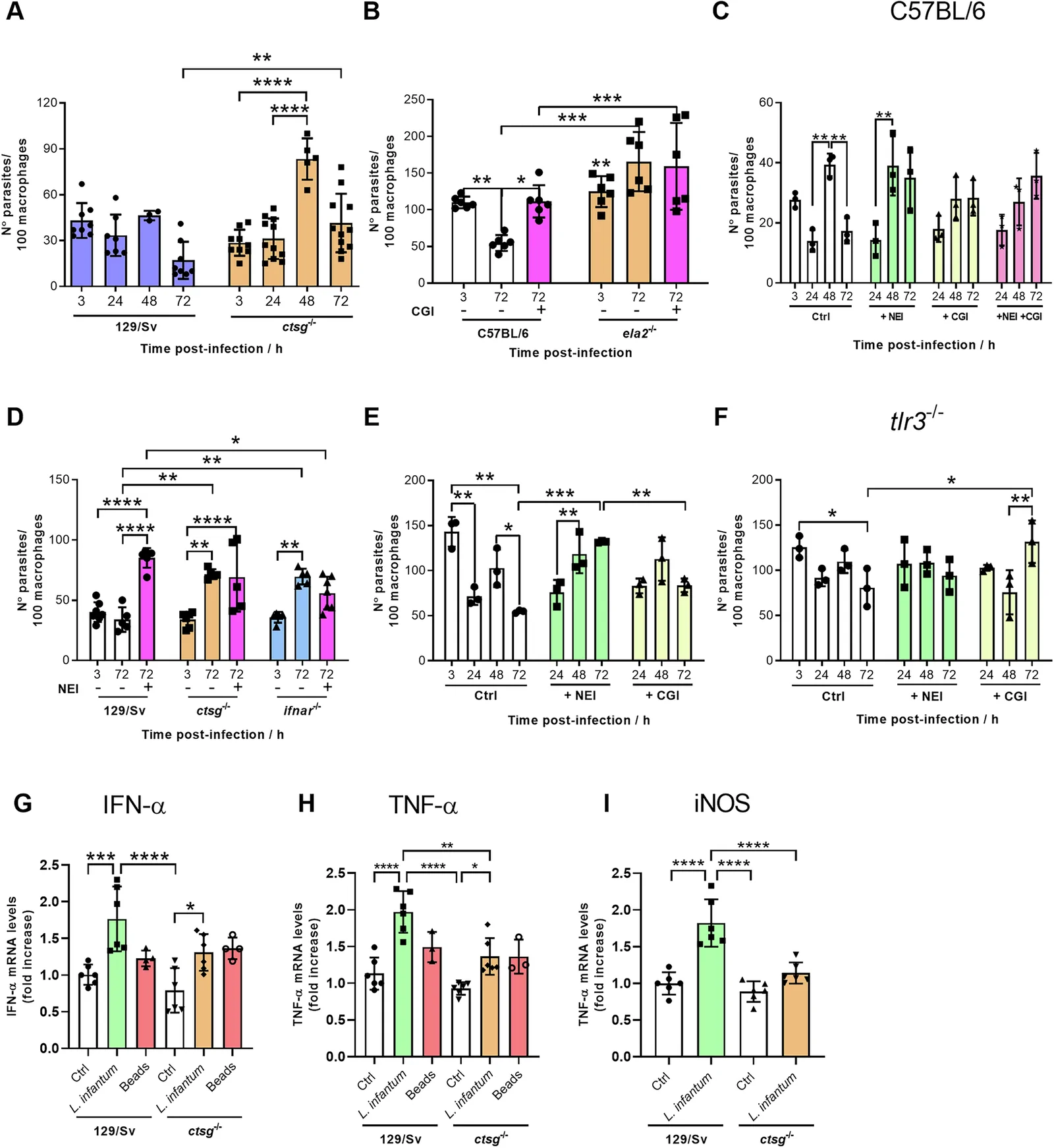
Neutrophil elastase and cathepsin G are required to enable parasite killing. Peritoneal thioglycolate-recruited macrophages from **A**, **D** 129/Sv, cathepsin G-knockout (*ctsg*^-/-^) and **D** type I interferon receptor (IFNAR)-knockout mice (*ifnar*^-/-^), (**B**, **C**, **E**, **F**) C57BL/6, **B** neutrophil elastase-knockout mice (*ela2*^-/-^), or **F** Toll like receptor 3 (TLR3)-knockout mice (*tlr3*^-/-^) were cultivated on glass coverslips overnight in RPMI-FCS then washed and infected with promastigotes of *L. infantum* at a 5:1 parasite:macrophage ratio for at 37 °C in RPMI-BSA. After 3 h, the monolayers were washed with BSS to remove extracellular parasites, fixed with methanol, and Giemsa-stained, or further incubated for the indicated times in RPMI-FCS at 37°C before fixation and staining. The number of intracellular parasites was determined under the light microscope. In (**C, E**, **F**), the reversible cathepsin G inhibitor I (CGI; 10 µM; Calbiochem) was added 5 min prior to infection of the macrophages. Where indicated, in (**C**, **E**, **F**) the irreversible inhibitor of NE, MeOSuc-AlaAlaProVal-CMK (NEI; 10 µM; Calbiochem) was added 5 min prior to infection of macrophages. **A**, **B**, **D** Experiments were performed three or four times (with 3 replicates each or 2 replicates each). Graphs show means ± SD of biological replicates compiled. Statistical significance was assessed using two-way ANOVA with **A** only Sidak’s, or **B**–**E** both Tukey’s and Sidak’s multiple comparisons tests. **G**–**I** Thioglycolate-recruited macrophages from 129/Sv and from *ctsg*^-/-^ mice were infected with promastigotes of *L. infantum* at a 10:1 parasite: macrophage ratio in RPMI-BSA at 37 °C for 2 h. Cultures were then washed and incubated in RPMI-BSA for a further 4 h, after which cells were washed and total RNA was extracted. cDNA was made and used as templates in qPCR for the determination of the relative mRNA levels for **G** IFN-α, **H** TNF-α and **I** iNOS. Uninfected cells were used as control (Ctrl) levels. Experiments were performed two times or three times. Graphs show means ± SD of biological replicates from independent experiments compiled. **A** *n* = 8, **B** *n* = 6, **C** *n* = 3, **D** *n* = 5, **E** *n* = 3, **F** *n* = 3, **G** n = 5, **H** *n* = 5, and **I** *n* = 6 biologically independent samples compiled from independent experiments. Statistical significance was assessed using one-way ANOVA with Bonferroni multiple comparisons test (alpha level of 0.05); **F**–**I** *P* < 0.0001. Significant differences between groups at the same timepoint/under the same treatment and between timepoints/treatments within the same group are indicated; *P < 0.05, **P < 0.01, ***P < 0.001, ****P < 0.0001. Asterisks over bars indicate significant differences compared to the data for all other timepoints or treatments within the groups.

Besides NE, cathepsin G (CG) is a S1A serine peptidase that is present in monocytes^10^, and we asked if it plays a role in infection by *L. infantum*. We observed that macrophages derived from CG-knockout mice (*ctsg*^-/-^) were more permissive to the proliferation of *L. infantum* at 48–72 hours as compared to macrophages from background mice (Fig. 9A). To test whether NE and CG acted through a common pathway, we used a CG inhibitor in infections of macrophages from *ela2*^-/-^ and respective C57BL/6 background mice. While the CG inhibitor had a mild effect in increasing parasite burdens at 72 hours in C57BL/6 macrophages, it did not further increase parasitism in *ela2*^-/-^ macrophages (Fig. 9B). This suggests that in C57BL/6 macrophages, although both S1A peptidases are required to control infection by *L. infantum* they do not seem to have additive effects. This was further tested in C57BL/6 macrophages by checking if both NEI and CGI would display an additive effect in sustaining parasite burden at 72 h (Fig. 9C). The addition of each inhibitor separately or in conjunction exerted a similar effect in sustaining parasite burden at 72 h, favoring the conclusion that both enzymes do not act additively but rather in common pathways in C57BL/6 macrophages. We further verified the effect of the NE inhibitor in macrophages of CG knockout mice. Inhibition of NE increased parasite burdens at 72 h in 129/Sv (Fig. 9D). In *ctsg*^-/-^ macrophages, results were variable, and there was no clear increase in the burden at 72 h. This supports the notion that NE and CG do not have additive roles to enable parasite killing. Furthermore, we observed increased parasite burdens at 72 h in *ifnar*^-/-^ macrophages (Fig. 9D), confirming that *L. infantum* infection is negatively controlled by type-I IFN. 129/Sv macrophages likely kill *L. infantum* through IFN-α, whose levels are induced upon infection, are sufficient to prevent growth at 72 h, and this requires CG. Given that *L. infantum* was unable to colonize macrophages from TLR3-knockout mice, we asked whether this was a consequence of NE- or CG-mediated pathways. Inhibition of either peptidase promptly enhanced parasitism of C57BL/6 macrophages (Fig. 9E) but, conversely, was ineffective in restoring parasite burdens in *tlr3*^-/-^ macrophages (Fig. 9F). This suggests that although TLR3 contributes to induction of IFN-α and TNF-α via NE and CG, that promote killing, this alone cannot account for the lack of parasite growth in 72 h in the absence of TLR3, and likely additional molecules and/or pathways downstream of TLR3 are necessary to support *L. infantum* infection. The contribution of CG for the induction of gene expression was then addressed by qPCR. We found that the induction of IFN-α, TNF-α, and iNOS (Fig. 9G-I) in infected macrophages was dependent on CG. Even though IFN-α and TNF-α were induced in macrophages deficient in CG (Fig. 9G-H), the levels were like those provoked by inert beads, suggesting that, at least to some extent, IFN−α induction is dependent on CG, while it was completely abolished in the absence of NE. This indicates that both NE and CG contribute directly to the control of *L. infantum* in macrophages through the induction of pro-inflammatory cytokines, such as TNF-α and presumably NOS, while NE is a major contributor to the induction of IFN-α.

**Fig. 10:**
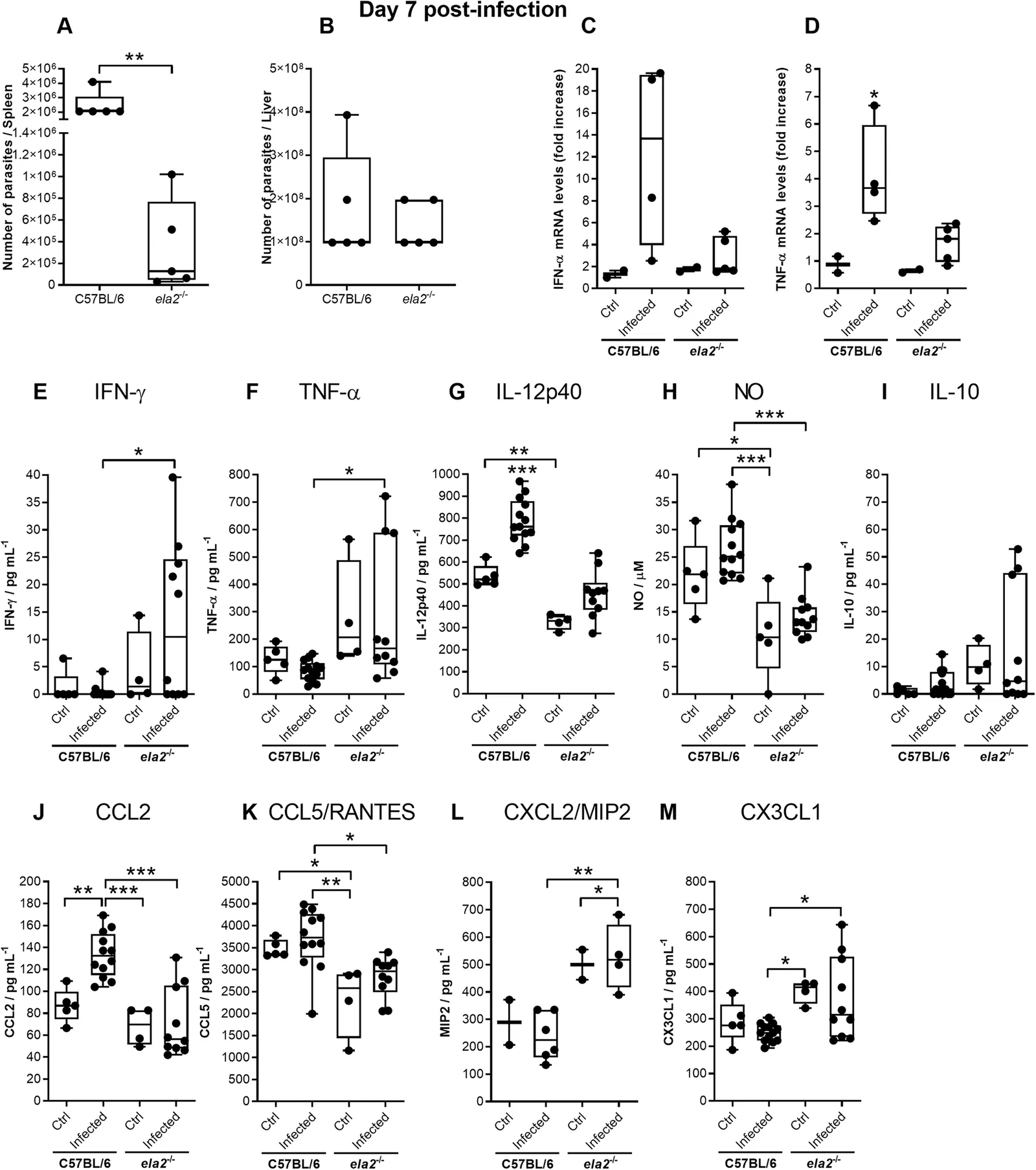
Neutrophil elastase is required for *L. infantum* to establish infection in vivo. C57BL/6 and neutrophil elastase-knockout (*ela2*^-/-^) mice were infected by retro-orbital inoculation of *L. infantum* promastigotes (10^7^ parasites/animal in PBS). After 1 week, the spleens and livers were removed, a sample was taken for RNA extraction, and the rest was homogenized. Parasite loads in (**A**) spleen and (**B**) liver were assessed by limiting dilution and represented as parasites/organ. Graphs show 2 independent experiments compiled (2 times n = 5 animals/group, each time). Each point represents an individual mouse and graphs are shown as box and whisker plots with the median (center line), lower and upper quartiles (25th and 75th percentiles; box limits), and min/max (whiskers). Statistical analyses were performed using an unpaired *t*-test; **A** *P* = 0.0016 and **B** *P* = 0.5460. **C**, **D** Total RNA was extracted from spleen samples, reverse transcribed to cDNA, which was used as templates in qPCR for the determination of the relative mRNA levels for **C** IFN-α and **D** TNF-α. The homogenized spleens were centrifuged, and the supernatants were collected for determination of cytokine levels by ELISAs: **E** IFN-γ, **F** TNF-α, **G** IL-12p40, **H** nitric oxide (NO), **I** IL-10, **J** CCL2, **K** CCL5, **L** CXCL2, and **M** CX3CL1. Each point represents an individual mouse, and graphs show the combined results of 2 independent repeats, except for **M**, which shows the data from one independent experiment. Uninfected mice were used as a control (Ctrl). **C**–**M** Statistical significance was assessed using one-way ANOVA with Tukey’s multiple comparisons test (alpha level of 0.05). Significant differences between groups are shown over lines, while asterisks over plots indicate significant differences compared to all other groups; *P < 0.05, **P < 0.01, ***P < 0.001.

The contribution of host SPs for the initial establishment of parasite infection in the target organs was subsequently verified in the experimental mouse model infected with *L. infantum* promastigotes. In the absence of NE, at day 7, parasite burdens in the spleen were significantly reduced as compared to those of the background C57BL/6 mice (Fig. 10A; *P* = 0.0016), while they were similar in the infected livers (Fig. 10B; *P* = 0.5460). These observations were discrepant with the leishmanicidal effect of NE for macrophages in vitro. However, the gene expressions of IFN-α and TNF-α (Fig. 10C, D) were reduced in the spleens of infected *ela2*^-/-^ mice, as compared to those of infected C57BL/6 mice, mirroring the findings in infected macrophages in vitro. The analysis of main inflammatory cytokines at the protein level revealed that the microenvironment in the spleens of infected *ela2*^-/-^ mice differed from that of C57BL/6 mice in a variety of ways. In the absence of NE, there were significant levels of IFN-γ and TNF-α (Fig. 10E, F) in the spleen, which were significantly increased when compared to infected C57Bl/6 mice. Even though the baseline levels in uninfected knockout mice are generally higher than in C57Bl/6 mice, no statistical significance was found between those groups. In contrast, IL-12 and NO levels (Fig. 10G, H) were elevated in infected C57Bl/6 mice as compared to uninfected controls. In contrast, in mice deficient in NE, infection does not induce IL12 or NO, suggesting a role for this peptidase in the accumulation/induction of those inflammatory molecules during early stages of infection by *L. infantum*. Furthermore, there was no statistical significance for the levels of IL-10 among the groups (Fig. 10I). The pattern of chemokines was also verified to aid in the prediction of cellular subtypes that could be recruited to the spleen at the initial stages of infection. The protein levels of all tested chemokines in the spleens differed between infected C57BL/6 and *ela2*^-/-^ mice, as compared to their respective uninfected controls. While infection induced high levels of CCL2 and CCL5 (also known as RANTES) in C57BL/6 mice (Fig. 10J, K), those remained unchanged in *ela2*^-/-^ mice upon infection, revealing that NE is required to induce those chemokines in *L. infantum*-infected mice. CCL2, but not CCL5, is involved in the early recruitment of monocytes^16^, which could increase the delivery of potential host cells for *Leishmania* to the spleen. In vivo infection did not induce changes in CXCL2 (also known as MIP-2) nor CX3CL1 in the spleen (Fig. 10L, M), as compared to uninfected controls. However, *ela2*^-/-^ mice contained higher background levels of those chemokines compared to C57BL/6 mice, which could affect the baseline composition of the cellular subpopulations in the spleens of the knockout mice.

**Fig. 11:**
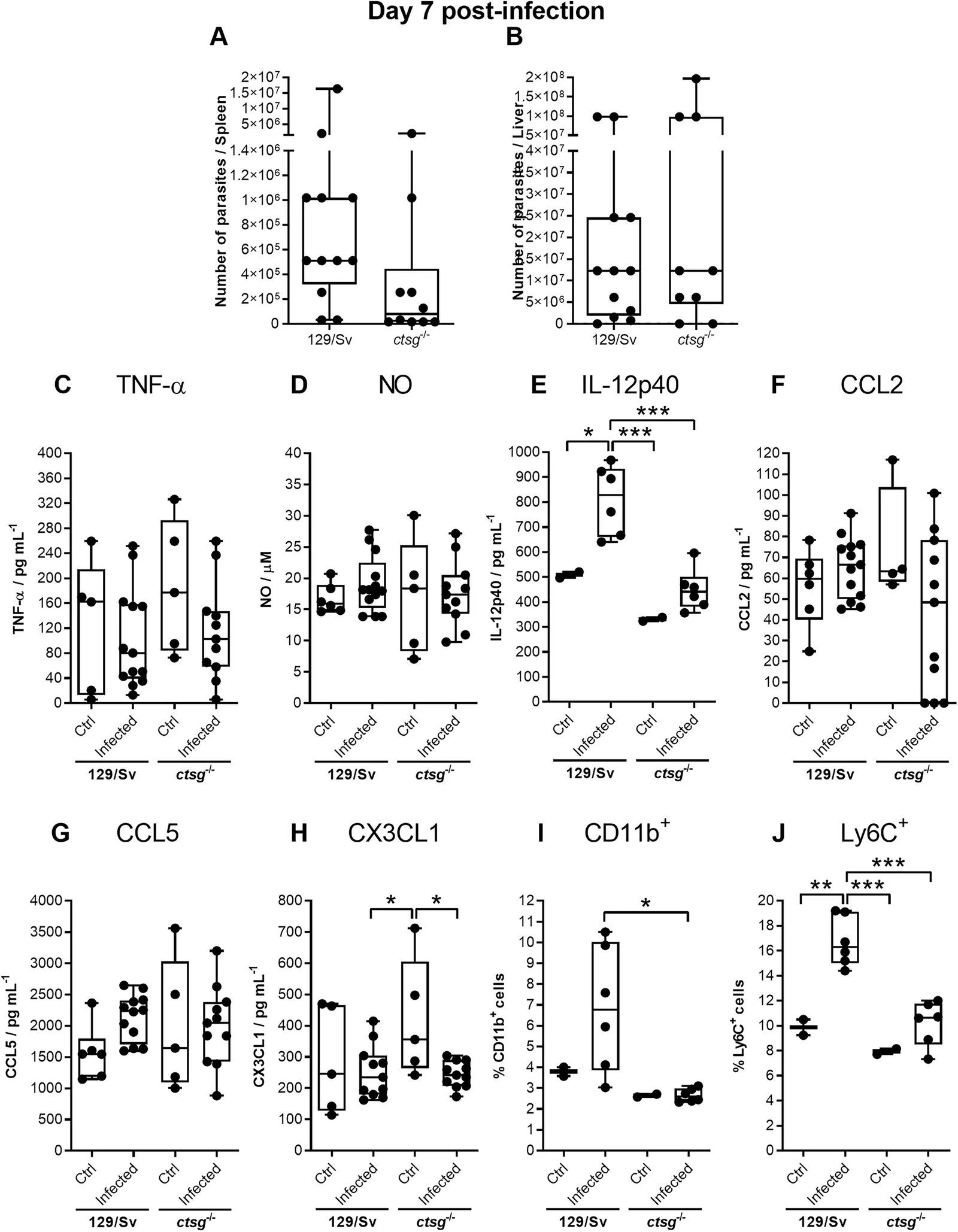
Cathepsin G is required for the early recruitment of innate myeloid cells to the spleen. 129/Sv and cathepsin G-knockout (*ctsg*^-/-^) mice were infected by retro-orbital inoculation of *L. infantum* promastigotes (10^7^ parasites/animal in PBS). After 1 week, parasite loads in (**A**) spleens and (**B**) livers were homogenized and assessed by limiting dilution and represented as parasites/organ. Graphs show the combined results of 2 independent experiments (2 times *n* = 12 for 129/Sv and *n* = 10 each time for *ctsg*^-/-^). Each point represents an individual mouse, and graphs are shown as box and whisker plots with the median (center line), lower and upper quartiles (25th and 75th percentiles; box limits), and min/max (whiskers). Statistical analyses were performed using an unpaired t-test. The homogenized spleens were centrifuged, and the supernatants were collected for determination of cytokine levels by ELISAs, and the cell suspensions were stained with antibodies to determine leukocyte subpopulations by flow cytometry. Cytokine levels were determined for **C** TNF-α, **D** nitric oxide (NO), **E** IL-12p40, **F** CCL2, **G** CCL5/RANTES, and **H** CX3CL1. Graphs show the combined results of 2 independent experiments. **I**, **J** The total CD11b^+^ cells in the spleen and Ly6C^hi^ cells within the CD11b^+^ population were determined by flow cytometry. Uninfected mice were used as a control (Ctrl). Results are presented as box and whisker plots with the median (center line), lower and upper quartiles (25th and 75th percentiles; box limits), and min/max (whiskers). Statistical analysis was performed using one-way ANOVA with Tukey’s multiple comparisons test (alpha level of 0.05). Significant differences between groups are indicated; *P < 0.05, **P < 0.01, ***P < 0.001.

Infections of *ctsg*^-/-^ mice revealed a similar pattern to that observed in *ela2*^-/-^ mice, with reduced parasite burden in the spleens (Fig. 11A), as compared to their genetic background, but similar burdens in the liver (Fig. 11B). In contrast, the levels of TNF-α and NO (Fig. 11C, D) were nearly similar in the spleens among the groups, irrespective of infection and/or genetic background, the exception being IL-12 (Fig. 11E), which was induced upon infection in 129/Sv but not in *ctsg*^-/-^ mice. IFN-γ and IL-10 levels were nearly undetectable (Supplementary Fig. 4). Likewise, protein levels of CCL2 and CCL5 (Fig. 11F, G) were similar among groups, while baseline levels of CX3CL1 were higher in *ctsg*^-/-^ mice, as compared to their genetic background (Fig. 11H), in common with *ela2*^-/-^ mice. Significant changes in the infiltration of the mononuclear subsets in the spleens of infected 129/Sv mice were observed, as shown by higher percentages of CD11b^+^ cells (Fig. 11J, gating strategy shown in Supplementary Fig. 5), more specifically, of the Ly6C^hi^ subset (Fig. 11K). In contrast, in *ctsg*^-/-^ mice, the percentages of those cellular subsets remained unchanged upon infection, revealing the requirement of this SP for early recruitment of innate myeloid cells to the spleen. This is in line with current knowledge that CG activity regulates the inflammatory response and the mobilization of cellular recruitment to damaged and/or infected tissues in many settings^17^. The main lymphocyte subsets were generally similar among the groups (Supplementary Fig. 4), with a slightly higher percentage of CD4^+^ lymphocytes in infected *ctsg*^-/-^ mice.

**Fig. 12:**
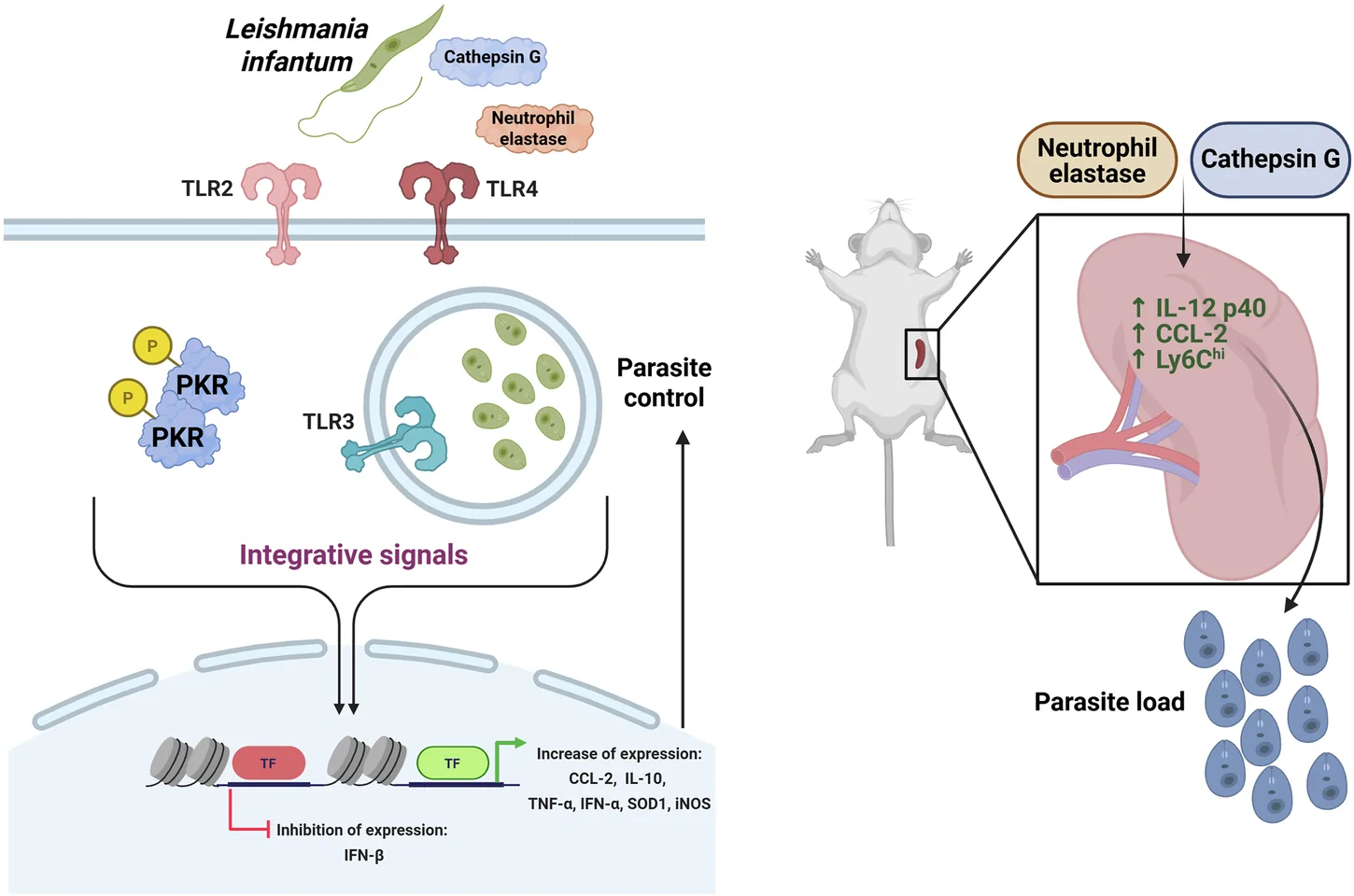
Modulation of *L. infantum* infection by neutrophil elastase and cathepsin G. During the infection of inflammatory primary murine macrophages by *L. infantum*, neutrophil elastase (NE) acts in conjunction with TLR4 to induce the expression of pro-inflammatory IFN-α, TNFα, iNOS, and of anti-inflammatory IL-10 and SOD-1, but not of IFN-β, within 6 h. IFN-α and TNF-α contribute to control intracellular parasitism at day 3 post-infection. TLR2 also contributes to control cellular parasitism and to provoke expression of TNFα and iNOS, but contributes to a less extent to the induction of IFN-α. TLR3 contributes to the expression of IFN-α and TNFα. Nevertheless, TLR3 and protein kinase R (PKR) are required for sustained macrophage parasitism by *L. infantum*. Cathepsin G is also required to promote the expression of IFN-α, TNF-α, and iNOS in infected macrophages, and acts in a common pathway with NE. In mice of the C57Bl/6 background, at the initial stage of infection (day 7), NE contributes to elevated mRNA of IFN-α, TNFα, and to the production of CCL-2, IL-12, and nitric oxide (NO) in the spleen. Despite the less inflammatory spleen environment in the absence of NE, the enzyme contributes to promote parasite burden in vivo. In mice of the 129/Sv background, cathepsin G did not contribute to the levels of TNF-α, CCL-2, and NO, but was required for the production of IL-12 and CCL-2, as well as for the recruitment of myeloid cells, especially that of Ly6^+^ monocytes.

Taken together, our results show that the same molecular machinery utilized by *L. donovani* and *L. infantum* can have opposite consequences to parasite fate at the cellular level. While both NE and CG exert a prominent role in controlling parasite burdens in macrophages via IFN-α and TNF-α, these enzymes contribute positively to the initial survival of *L. infantum* in the spleens of mice, likely due to changes in the balance of a network of infiltrating cellular subsets and cytokines, which could favor the recruitment of cells that contribute to initial parasitism. The conclusions are summarized in the schematic representation (Fig. 12).

## Discussion

The diverse species of *Leishmania* are finely adapted to survive and proliferate in macrophages, and have evolved different strategies to modulate the host cell response. We have previously described a mechanistic pathway dependent on the proteolytic activity of NE that acts distinctively to promote or control infection, depending on the *Leishmania* species^5,6,8,9,18^. In common, NE present in macrophages requires TLR4 to induce responses upon infection, but the outcome varies with the *Leishmania* species. While in *L. major* infection, NE activity induces TNF-α and both type I IFNs, IFN-α and IFN-β, leading to parasite killing, in *L. donovani*-infected macrophages, high levels of NE-induced IFN-β are crucial for parasite growth. This is in line with other reports showing that in *L. amazonensis* infections, TLR2-induced IFN-β is likewise essential for parasite proliferation and sustained intracellular burdens^4,15^. In common, the induction of type I IFNs is a central response in infected macrophages, and *Leishmania* has adapted to either avoid or allow it to take place. *L. major* inhibits NE activity by its endogenous inhibitor ISP2, while *L. donovani* does not express ISP2 as an adaptation to engage the TLR-NE pathway required for growth. Here, we found that the responses induced in macrophages by *L. infantum* are remarkably different from those observed with *L. donovani*, suggesting that the two visceralizing species have evolved alternative ways to survive in macrophages. Even though we have previously shown that in chronic infection in BALB/c mice with either *L. donovani* (LV9 strain) or *L. infantum*, the host immunity-related genes induced at the mRNA level are similar, we did not dissect how specific pathways could directly contribute to infection of the host cell^19^. Remarkably, *L. infantum* was less adapted than *L. donovani* to modulate antiparasitic responses of macrophages at the cellular level. *L. infantum* induced high expression of iNOS and was less capable than *L. donovani* to induce SOD1, suggesting that *L. infantum* is less efficient in controlling oxidative stress, at least so in thioglycolate-primed macrophages. IL-10 is a main cytokine to act on macrophages to render them permissive of *Leishmania*^20^, and mice lacking IL-10 are highly resistant to *L. donovani* infection^21^. The fact that high levels of IL-10 mRNA (nearly 20-fold increase) were induced in macrophages by *L. infantum* argues against variations in IL-10 being the main reason for the increased signs of oxidative stress in *L. infantum*-infected macrophages. It remains to be determined if this premise also applies to Kupffer cells, which are the host cells for visceral *Leishmania* in active liver infection.

In common with *L. major*, we found that engagement of the NE-TLR axis by *L. infantum* induces pro-inflammatory cytokines that are detrimental for parasite growth. More specifically, type I IFNs are able to kill intracellular parasites, and the levels induced by *L. infantum* were sufficient to control parasitism, as shown by increased parasite loads in macrophages from IFNAR-knockout mice. Of note, we found that IFN-β was irrelevant to *L. infantum* capability to colonize macrophages, i.e., besides not being induced, exogenous IFN-β did not affect parasite burdens in host cells, denoting a main difference between the adaptations of *L. donovani* and *L. infantum*. Considering that both IFN-α and IFN-β act through IFNAR, one could expect that both cytokines would exert an effect in intracellular parasitism. However, differences in receptor binding affinity for the different type I IFNs and the number of surface receptor molecules available for binding determine different activities of the individual IFNs^22^. In addition, controlled receptor endocytosis is also a major player in the regulation of the signal transduction of IFNAR and of the downstream gene expression profile^23^. In that sense, exogenously added IFNs may have induced different signaling outcomes with consequences to parasitism, resulting in effective parasite control via IFN-α, but not via IFN-β. It is intriguing that while *L. donovani* induced a detectable increase in the expression of both IFN-α and IFN-β^6^, we only observed increases in IFN-α in *L. infantum*-infected cells. This pattern was repeatedly observed in infected macrophages, which could otherwise induce IFN-β upon stimulation with poly I:C, showing that the macrophages used in our infection model did not have an impairment in the expression of IFN-β. While there is one IFN-β gene, there are multiple IFN-α genes that can be grouped in clusters and vary in number according to the species, there being 14 IFN-α genes in the mouse^24^. Since we used degenerate primers to detect all IFN-α subtypes, we cannot discriminate if a specific IFN-α gene, or which subtypes, are being transcribed in response to *L. infantum*. While expression of IFN-β can be achieved in isolation, the expression of IFN-α genes is usually preceded by and enhanced by IFN-β^25^, which was not the case in *L. infantum*-infected macrophages. Alternatively, a subtle amount of IFN-β that is below detection in our methods could be induced, helping to increase expression of IFN-α via an autocrine loop. IFN-β expression is regulated by transcription factors that bind to four regulatory promoter domains, the main factors being IRF3 and IRF7. Although the promoter domains for the IFN-β gene and for the different IFN-α genes share 95% identity, there are several insertions and three short deletions in the promoters of the different IFN-α genes, as compared to that of IFN-β, that alter their binding to IRFs. Such differences could help to explain why *L. infantum* induced IFN-α expression, but not IFN-β, at least at the timepoints used in this study. The current model proposes differential regulation of IFN-α genes by activation of either IRF3 or IRF7, or co-activation of both, depending on their protein levels^22,26^. We have shown that *L. donovani* induces the translocation of IRF3 to the nucleus of the infected macrophage^6^. In a different infection model using RAW cells and *L. donovani* 8G83, Saha et al. reported reduced phosphorylation of IRF3 at early infection (15-60 min), as compared to cells stimulated with LPS, even though IRF3 phosphorylation still took place at 120 min post-infection^27^. However, the degree of IRF3 translocation to the nucleus was not addressed. Infected RAW cells also exhibited elevated levels of heme oxygenase, whose heme degradation byproducts correlated with reduced TNF-α, NFkB, and IRF3 phosphorylation. Although not addressed here, it is possible that differences in the balance of IRF3 and/or IRF7 levels in the *L. infantum*-infected cell also contribute to the selective increase in mRNA for IFN-α.

Previous work showed that *L. infantum* induces IFN-β mRNA in bone marrow-derived dendritic cells (DCs) via TLR4, and that this receptor was central to regulate the transcriptional profile of infected DCs^28^. Although we have not addressed the pattern of gene expression of DCs in our work, we did not observe IFN-β induction in primary macrophages, suggesting that engagement of TLR4 by *L. infantum* can have different consequences for innate and adaptive immunity, depending on the cellular subtype. Optimal IFN-β induction by *L. donovani* required the additive roles of TLR2, presumably via lipophosphoglycan (LPG), and of TLR4 via NE^6^. Although the exact mechanisms through which IFN-β promotes macrophage colonization by *L. donovani* are unknown, an elegant study has connected the requirement of type I IFNs with mitochondrial biogenesis and parasite burden in macrophages^29^. In line with our findings, the authors found that both TLR2 and TLR4 were required for long-term parasitism in macrophages, denoting a positive role for those TLRs, and for downstream IFN-β, in the ability of *L. donovani* to grow in macrophages^29^. In line with opposite adaptations of the two visceralizing species, we found that both TLR2 and TLR4 contribute to control macrophage parasitism by *L. infantum*. While it is expected that TLR2 is promptly activated by *L. infantum* via LPG, by analogy to other *Leishmania* species, it is intriguing at first that the parasite engages the NE-TLR4 pathway, which drastically reduces long-term macrophage colonization. Although *L. infantum* could grow in inflammatory macrophages in vitro, this took place at an apparent suboptimal condition because parasite burdens were significantly improved by the inhibition or absence of NE or CG.

TNF-α and IFN-α showed high leishmanicidal activity against intracellular *L. infantum*, which lies well with the notion that TNF is leishmanicidal^30^. The expression of these cytokines was induced by NE and was fully dependent on TLR2 and TLR3, which was also the case for the induction of IFN-β by *L. donovani*^6^. This indicates that a common upstream signaling pathway is shared by both species, and suggests that the direction of the response diverges downstream of TLR3 and PKR. Downstream TLR3 signaling is TRIF-dependent, leading to a complex cascade that involves NF-κB, AP1, and IRF3 for the induction of multiple genes^30^. Since ubiquitination and phosphorylation of many signaling molecules of the TLR3 pathway are essential to tune the response^31^, it is feasible that *L. infantum* and *L. donovani* might differ in how they provoke alterations in the network of downstream players. While NE acts through TLR4, LPG is expected to be the main activator of TLR2^32^ and the crosstalk between both receptors could result from a converged single signaling platform at the cell surface, formed at the region of contact with the parasite. Indeed, we had shown that *L. major* induces the colocalization of NE and CD11b at the surface of macrophages^8^, while NE and TLR4 colocalize with *L. donovani*^5^. We reported that TLR3 activation by *L. amazonensis* involves a nucleic acid-independent pathway by direct proteolytic cleavage of the receptor^33^. Lack of TLR3 resulted in lower IFN-β and IL-10 and increased IL-12 in macrophages, which became less permissive to *L. amazonensis* growth^33^. Here, *L. infantum* did not induce IFN-β and was refractory to it, so its dependence on TLR3 might rely on other mechanisms. Considering that the induction of SOD1 was dependent on TLR3, it is possible that the absence of this enzyme to counteract oxidative stress in a condition that is already suboptimal for parasite growth renders macrophages fully resistant to infection.

We have also shown that cathepsin G contributes directly to control infection, and we had indication that it does not play an additive role to that of NE in macrophages in the C57BL6 background. Since the increase in IFN-α mRNA in *ctsg*^-/-^ macrophages was still greater than that in uninfected controls, whereas it was completely abolished in *ela2*^-/-^ macrophages, it is plausible to reason that while IFN-α induction depends mainly on the crosstalk between NE-TLR4 and LPG-TLR2, additional mechanisms could also contribute to CG-dependent leishmanicidal activity. CG was described as a pivotal enzyme determining pyroptosis in monocytes and neutrophils, and its inhibition increases cellular survival at steady-state levels^34^. In macrophages, CG was responsible for the cleavage of Gasdermin-D and increased release of inflammatory cytokines upon lipopolysaccharide challenge^34^. Noteworthy, Gasdermin-D was found as the activator of the NLRP3 inflammasome during *Leishmania* infection, being connected to host resistance^35^. In such a model, although the inflammasome was activated within 2 h of infection with *L. amazonensis*, the parasite prevented subsequent macrophage pyroptosis, and Gasdermin-D cleavage resulted in a noncanonical 25 kDa fragment. It remains to be tested whether *L. infantum* is also capable of downmodulating host cell death. Although we have not tested inflammasome activation, an attractive hypothesis is that the leishmanicidal role of CG involves Gasdermin-D-dependent inflammasome activation.

Finally, our in vivo experimental infections revealed that both NE and CG are fundamental for establishing early splenic infection, despite their paradoxically leishmanicidal role at the cellular level. We determined that NE is crucial for dampening early IFN-γ and TNF-α. This suppression may be critical for allowing infection to take hold while the adaptive immune response is still mounting, as these cytokines are potent activators of microbicidal M1-type macrophage responses. Conversely, mice lacking NE or CG failed to induce splenic IL-12 upon infection compared to their genetic background controls, suggesting that these proteases may participate in driving the early Th1 response. Notably, NE exhibited a strong pro-inflammatory profile in vivo, also mediating increases in NO and RANTES, and in the mRNA levels for IFN-α.

While the opposing roles of NE and CG in parasite survival in vitro and in vivo initially appear discrepant, our cellular recruitment data offer a plausible explanation for why *L. infantum* requires these proteases to establish parasitism by day 7. At this early time point, infection is likely still restricted to the initial wave of host cells, given that the full intracellular replication cycle of the parasite exceeds 5 days. We found that NE is crucial for the production of monocyte chemoattractant CCL2, while CG is required for the recruitment of Ly6^+^ myeloid cells. In line with this, Romano et al. demonstrated in a murine *L. major* infection model that inflammatory monocytes serve as the primary mononuclear hosts for initial parasite replication^36^. Therefore, by driving CCL2 production and subsequent myeloid cell recruitment, NE and CG inadvertently provide *L. infantum* with a continuous supply of highly susceptible host cells to sustain early splenic infection.

Here, the most noticeable differences in vivo were the lack of CCL2 in the infected spleens in the absence of NE. We confirmed that macrophages infected in vitro induced CCL2 mRNA, which was fully dependent on NE, even though in vivo, a broad range of cells can be the source of CCL2. Besides being the main chemoattractant of myeloid cells, CCL2 has a bigger role in leukocyte biology, impacting its behavior, polarization, autophagy, and survival^16^. In addition to recruiting T-cells, CCL2 can direct their maturation to the Th2 phenotype^37^, contributing to the modulation of infected tissue architecture. Noteworthy some of those other functions of CCL2 can depend on additional stimuli, and it is feasible to hypothesize that in the context of *L. infantum* infection, this cytokine plays a central role to enable parasitism, enabling recruitment of myeloid cells. Thus, preserving NE function in vivo could contribute to the production of adequate levels of CCL2, which in turn, adds to the recruitment of monocytes. Indeed, in a model of *L. major* infection, infiltrating permissive CCR2^+^ monocytes in the skin were pivotal in the establishment and the expansion of a replication niche for parasite^38^. A similar scenario could dictate initial infection in the spleen, where monocytes could be a primary source of permissive cells for *L. infantum*. This fits well with the increase in the Ly6C^hi^ population in the spleens of infected mice, which was absent from the spleens of infected *ctsg*^-/-^ mice. Notably, it has been reported that CG promotes monocyte chemotaxis directly via proteolytic activation of PAR-1^39^. Combined with CLL2, the proteolytic action of CG, resulting in chemotaxis of myeloid cells, could be crucial to maintain Ly6^+^ cellular infiltrates in the spleen. If so, this could help to explain why *L. infantum* has adapted to allow activation of macrophages by host SPs that, although resulting in cytokines that restrict macrophage colonization, at the same time, are crucial for the production of CCL2 and the recruitment of monocytes to the spleen. Together with other molecules, this could help to shape myeloid cell activation state and permissiveness to parasitism before adaptive immunity kicks in. In conclusion, we have identified SPs as the main pathway by which macrophages respond to infection by *L. infantum* and have established differences in the downstream mechanisms, as compared to the responses to *L. donovani*.

## Materials and methods

### Parasites

Wild-type promastigotes of the *L. donovani* MW897^5^ and *L. infantum* PP75 (MHOM/BR/1974/PP75) strains were grown in hemoflagellate modified Eagle’s medium (designated HOMEM medium) supplemented with 10% heat-inactivated fetal calf serum (FCS) (Gibco) at 27°C.

### Mice

The mouse strains used in this study are given in Table 1. The study received ethical approval from Comissão de Ética no Uso de Animais (CEUA) at the Universidade Federal do Rio de Janeiro. All mice were handled according to protocols approved by the ethics committee [Comissão de Ética no Uso de Animais (CEUA) 173/19-UFRJ]. The mice were kept in ventilated cages, in 12 h light/dark cycles, and provided with food and water *ad libidum*. For the in vivo experiments, male and female mice, aged between 8 and 14 weeks at the time of infection, were used (Table 1). For each independent experiment, the groups of different mouse strains were age- and sex-matched.

**Table 1:** Origin and background of mouse strains used in the study

Strain	Background	Origin	Colony
C57BL/6 J			Central animal facility at UFRJ
Neutrophil elastase (NE)-knockout (*ela2*^*-/-*^)	C57BL/6 J	Kindly donated by Dr. Bernhard Ryffel, Centre National de la Recherche Scientifique (CNRS), Orleans, France	Own colony maintained at LAT of UFRJ
Toll-like receptor 2 (TLR2)-knockout (*tlr2*^-/-^)	C57BL/6 J	^40^	Kindly donated by the colony of Dr. Marcelo T. Bozza (UFRJ)
Toll-like receptor 3 (TLR3)-knockout (*tlr3*^-/-^)	C57BL/6 J		Kindly donated from the colony of Dr. Renata Meirelles Pereira (UFRJ)
129/Sv			Own colony maintained at LAT of UFRJ
Protein kinase R (PKR)-knockout (*pkr*^-/-^)	129/Sv	Kindly donated by Dr. Aristóbolo M. Silva, Universidade Federal de Minas Gerais (UFMG), Minas Gerais, Brazil From^41^	Own colony maintained at LAT of UFRJ
Cathepsin G (CG)-knockout (*ctsg*^-/-^)	129/Sv	European Mouse Mutant Archive (EMMA)	Own colony maintained at LAT of UFRJ
IFN-α/β receptor alpha chain (IFNAR)-knockout (*ifnar*^-/-^)	129/Sv		Kindly donated by the colony of Dr. Herbert L. M. Guedes (UFRJ)

*LAT* Laboratório de Animais Transgênicos, *UFRJ* Universidade Federal do Rio de Janeiro.

### Macrophage infections

Peritoneal macrophages of mice were elicited by thioglycolate injection and collected as described previously^6^. For infection and survival assays, macrophages plated in 24-well culture plates containing coverslips (4 × 10^5^ cells/well) were infected with late-stage promastigotes (culture density of 2 × 10^7 ^mL^-1^) at a 5:1 parasite:cell ratio in RPMI 1640 medium (Sigma) supplemented with 0.1% bovine serum albumin (BSA; Sigma) at 37 °C and 5% CO_2_. After 3 h, cells were washed with Hank’s buffered saline solution (HBSS) to remove extracellular parasites. Macrophages were then either fixed with 70% methanol and Giemsa stained or cultured in RPMI-10% FCS for 24, 48, or 72 h and then fixed and stained. The number of intracellular parasites was determined by counting at least 100 cells/replicate under light microscopy at a 100× oil immersion objective. Where indicated in the figures, the TLR4 inhibitor (Resatorvid, synonym: TAK-242; 1 µM; MedChemExpress, catalog # Hy-11109) was added 15 minutes before the parasites. The irreversible NE inhibitor III, N-Methoxysuccinyl-Ala-Ala-Pro-Val-Chloro-methyl-ketone (MeMeOSuc-AAPV-CMK) referred to as NEI; 10 µM; Calbiochem, catalog #324245), or the reversible cathepsin G inhibitor I (10 µM; Calbiochem, catalog #219372) were added 5 min before the parasites, while IFN-α (200 ng mL^−1^; eBioscience, catalog # 4312049), IFN-β (200 ng mL^−1^; BioLegend, catalog # 581302), and TNF-α (200 ng mL^−1^; BioLegend, catalog # 575204) were added after the removal of extracellular parasites at 3 h and cultivated for 48 or 72 h in RPMI-10% FCS.

For RNA extraction, macrophages in 6-well plates (3 × 10^6^ cells/well) were infected with promastigotes at a 10:1 ratio in RPMI-0.1% BSA at 37 °C and 5% CO_2_. After 2 h, extracellular parasites were removed by washing with HBSS. Where indicated, polyinosinic-polycytidylic acid sodium salt (25 µg mL^−1^; poly I:C, Merck-Sigma, catalog # 31852-29-6) was added to macrophages alone, or macrophages were pre-treated with NEI (10 µM; Calbiochem) for 5 min prior to the addition of parasites. After removal of the parasites, cultures were incubated for a further 4 h in RPMI-0.1% BSA. Total RNA was extracted with the RNeasy Mini Kit (Qiagen) using a 1 mL syringe to initially lyse the cells.

### Quantitative PCR assays

The GoScript Reverse Transcriptase kit (Promega) was used to synthesize cDNA from total RNA. Real-time quantitative PCR (qPCR) assays of first-strand cDNA were performed using the GoTaq qPCR system (Promega), according to the manufacturer’s instructions, and the primers given in Table 2. Primers were synthesized by IDT (Integrated DNA Technology Inc.) and were used in prior work^6^. The qPCR reactions were performed at one cycle of 95 °C for 10 min, followed by 40 cycles of 15 seconds at 95 °C and 1 minute at 60 °C. The melting condition was 1 min at 60 °C at 0.1 °C per second up to 95 °C. Expression ratios were computed via the ΔΔCt method.

**Table 2:** Primers used for qPCR

Gene	Sequence
Interferon beta 1 (*Ifnb1*)	Sense 5′-TCCAAGAAAGGACGAACATTCG-3′ Antisense 5′-TGAGGACATCTCCCACGTCAA-3′
Interferon alpha (*Ifna* - all subtypes)	Sense 5′-CTGGCTGTGARRAMATACTTCC-3′ Antisense 5′-TTCTGCTCTGACMACCTCC-3′
Tumor necrosis factor (*Tnf*)	Sense 5′-GGTCCCCAAAGGGATGAGAAGTTC-3′ Antisense 5′-CCACTTGGTGGTTTGCTACGACG-3′
Nitric oxide synthase 2, inducible (*Nos2*)	Sense 5′-CAGCTGGGCTGTACAAACCTT-3′ Antisense 5′-CATTGGAAGTGAAGCGTTTCG-3′
Superoxide dismutase 1, soluble (*Sod1*)	Sense 5′-GGCAAGCGGTGAACCAGTTGTGTT-3′ Antisense 5′-TGAGGTCCTGCACTGGTACAGCC-3′
Interleukin 10 (*Il10*)	Sense 5′-CCCAGAAATCAAGGAGCATT-3′ Antisense 5′-TCACTCTTCACCTGCTCCAC-3′
2’-5’ oligoadenylate synthetase-like 2 (*Oasl2*)	Sense 5′-CAAGAAGTCAGGGTGATTAAGG-3′ Antisense 5′-GAACAGAATCATGTCTTGGTCA-3′
Glyceraldehyde 3-phosphate dehydrogenase (*Gadph*)	Sense 5′-TGCACCACCACCTGCTTAGC-3′ Antisense 5′-GGCATGGACTGTGGTCATGAG-3′

### In vivo infections

Stationary-phase promastigotes, 10^7^ in 100 µl PBS, were injected in the retro-orbital cavity of mice. After 1 or 5 wk, mice were euthanized, and the spleens and livers were collected. A sample of each organ (5 mg) was preserved in RNAlater™ (Thermo Fisher Scientific) until processing; samples were processed using a T 10 basic ULTRA-TURRAX® (IKA), and RNA was isolated using the RNeasy Mini Kit (Qiagen). The spleens were ruptured to homogeneity with the back of a syringe through a nylon membrane or cell strainer in RPMI medium (Thermo Fisher Scientific) supplemented with 10% FCS as described^6^, aliquots of the homogenates were submitted to 2-fold serial dilutions in 96-well plates, which were cultivated for 7 d at 27°C for parasite burden analysis; the highest dilution well was used to estimate the number of parasites multiplied by the dilution factors. The remaining homogenate volumes were centrifuged at 300×g for 5 min and the supernatants were collected and kept at -20°C for cytokine analysis. T n-sample calculation for a two-tailed t-test for independent samples was performed on the website powerandsampesize.com (http://powerandsamplesize.com/Calculators/Compare-2-means/2-Sample-Equality). The following parameters were applied: Statistical power 90%; Statistical significance threshold p < 0.05; Estimated difference between groups 20%; expected standard deviation 15%; Sampling ratio: 1 (considering comparison of groups with the same n).

### ELISAs

TNF-α, IFN-γ, IL-10, IL-12p40, CCL2, CX3CL1, CXCL2 (also known as MIP-2), and CCL5 (also known as RANTES) concentrations in the spleen supernatants were evaluated using R&D Systems DuoSet® kits. The nitric oxide concentrations were evaluated by the Griess reaction assay as described previously^9^. Plates were read on an Emax® Plus microplate reader (Molecular Devices).

### Flow cytometry

ACK-treated single-cell suspensions from the spleen (10^7^ cells) were washed in PBS at 300 × *g* for 5 min, incubated with an anti-Fc-γ III/II (CD16/32) receptor antibody (BioLegend) for 10 min, then stained with fluorochrome-conjugated antibodies for 30 min: BV421-CD11b (BioLegend, catalog # 101236), BV605-Ly6C clone HK1.4 (BioLegend, catalog # 128031), FITC-CD4 clone GK1.5 (eBioscience, catalog # 11-0041-85), PE-Cy5-CD8 clone 53-6.7 (eBioscience, catalog # 15-0081-83), and APC-CD19 clone MB19-1 (eBioscience, catalog # 17-0191-82). Cells were washed in PBS, fixed in 100 µl IC Fixation Buffer (eBioscience) for 30 min, then washed and resuspended in PBS. One hundred thousand events were acquired on a FACSCanto™ II (BD Biosciences), and data were analyzed on FlowJo v10. Gating strategies are described in Supplementary Figs. 4 and 5.

### Statistics and reproducibility

Statistical analyses were performed using Prism 7.0 (GraphPad Software). The data were analyzed as described in the figure legends, and the number of technical or biological replicates used to generate the graphs and the number of independent assays or experiments performed yielding similar trends between groups are also given in the figure legends. As variation in absolute numbers occurred between independent assays involving peritoneal thioglycolate-recruited macrophages, possibly owing to different activation states at each collection, statistical analysis was performed using technical replicates for each group in each independent assay.

### Reporting summary

Further information on research design is available in the Nature Portfolio Reporting Summary linked to this article.

## Supplementary information

Supplementary Information

Description of Additional Supplementary Files

SUPPLEMENTARY DATA

Reporting Summary

## Data Availability

All data supporting the findings of this study are available within the paper and its Supplementary Information. Raw data used to generate all the graphs in the manuscript have been submitted as a single Supplementary Data 1 excel file.
