# Supplementary material for: Cathepsin G and neutrophil elastase control Leishmania infantum parasitism through TLRs-dependent induction of IFN-α and TNF-α
Source: Commun Biol. 2026 Jun 29;9:1373. doi: 10.1038/s42003-026-10555-8 (PMC13683408; doi:10.1038/s42003-026-10555-8)
Supplement: Supplementary file 1 [file 42003_2026_10555_MOESM1_ESM.pdf]

Supplementary Figure 1

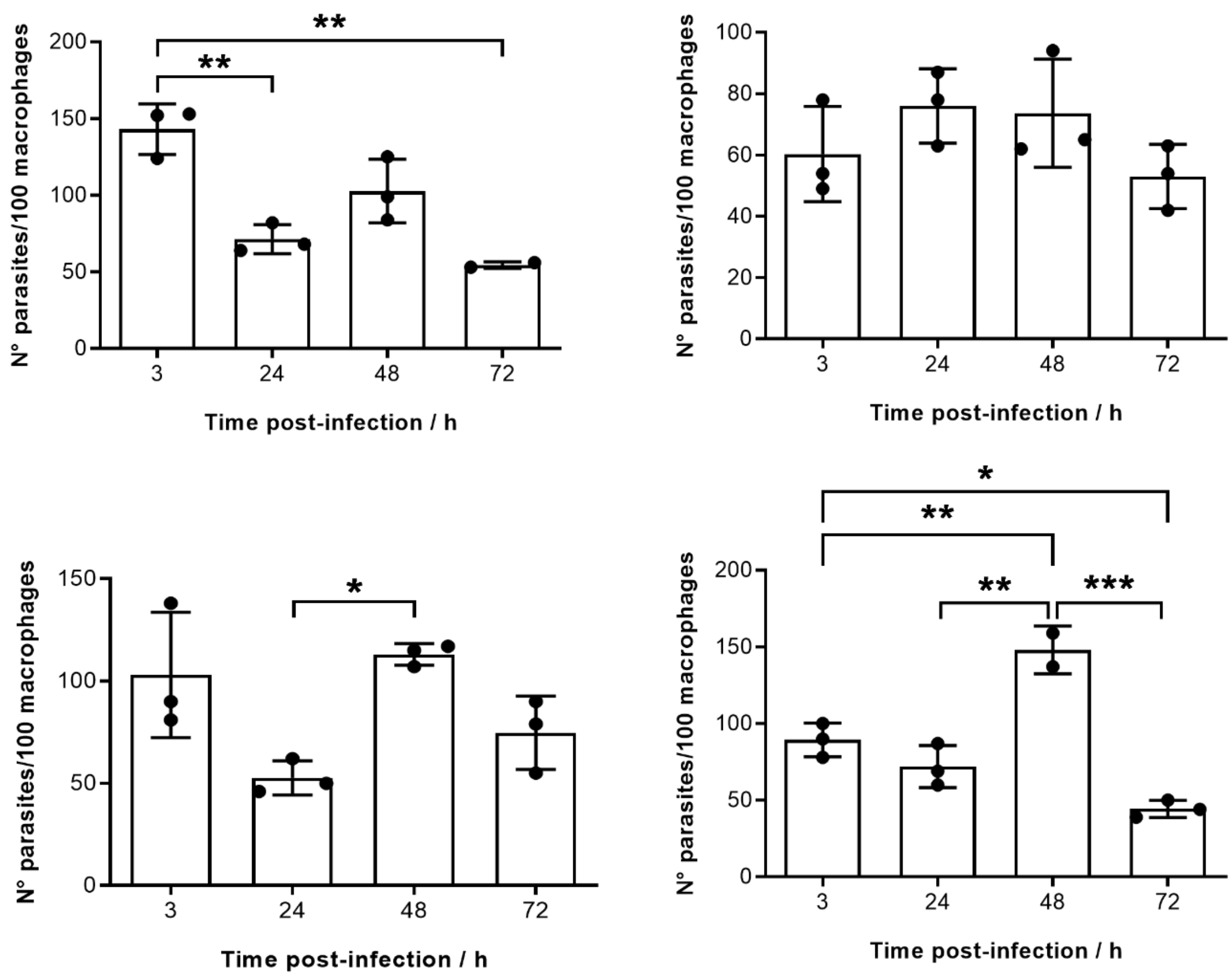


**Supplementary Fig. 1**: **Time-course of macrophage infection by *L. infantum*.** Peritoneal thioglycolate-recruited macrophages from C57BL/6 mice cultivated on glass coverslips overnight were infected with promastigotes of *L. infantum* at a 5:1 parasite:macrophage ratio in RPMI-BSA at 37°C. After 3 h, the monolayers were washed to remove extracellular parasites, fixed with methanol and Giemsa-stained, or further incubated for the indicated times in RPMI-FCS at 37°C before fixation and staining. The number of intracellular parasites was determined under the light microscope. N = 4. Each graph shows one experiment with three biological replicates. Asterisks indicate statistical significance at * P>0.05, **P <0.01, ***P <0.001. In common in all experiments, there was no parasite growth in macrophages at 72h post-infection.

Supplementary Figure 2

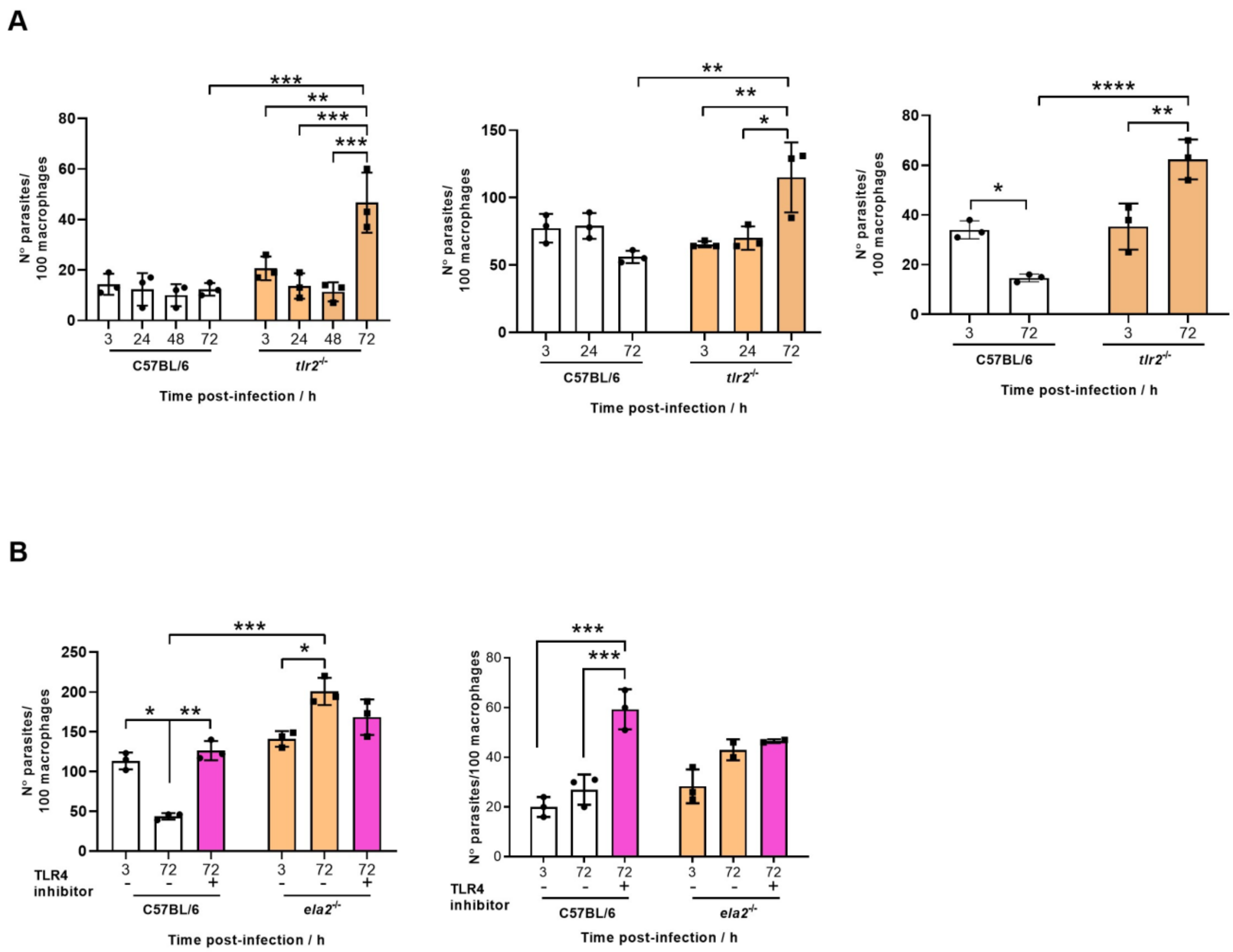


**Supplementary Fig. 2**: **TLRs in macrophage infection by *L. infantum*. (A)** Peritoneal thioglycolate-recruited macrophages from C57BL/6 mice and from Toll-like receptor 2 (TLR2)-knockout (*tlr2*$^{-/-}$) were cultivated on glass coverslips overnight and then infected with promastigotes of *L. infantum* at a 5:1 parasite:macrophage ratio in RPMI-BSA at 37°C. After 3 h, the monolayers were washed to remove extracellular parasites, fixed with methanol and Giemsa-stained, or further incubated for the indicated times in RPMI-FCS at 37°C before fixation and staining. The number of intracellular parasites was determined under the light microscope. Three independent experiments with 3 biological replicates each. Asterisks indicate statistical significance at $p>0.01$. In common in all experiments, there is parasite growth in macrophages of TLR2 knockout mice at 72h post-infection. **(B)** Peritoneal thioglycolate-recruited macrophages

from C57BL/6 mice and infected as described in (A). Where indicated, macrophages were pretreated with the inhibitor of TLR4, Restorvid (TAK-242; 1 nM; eBioscience) for 15 min prior to infection. Two independent experiments, with 3 biological replicates each.

Supplementary Figure 3

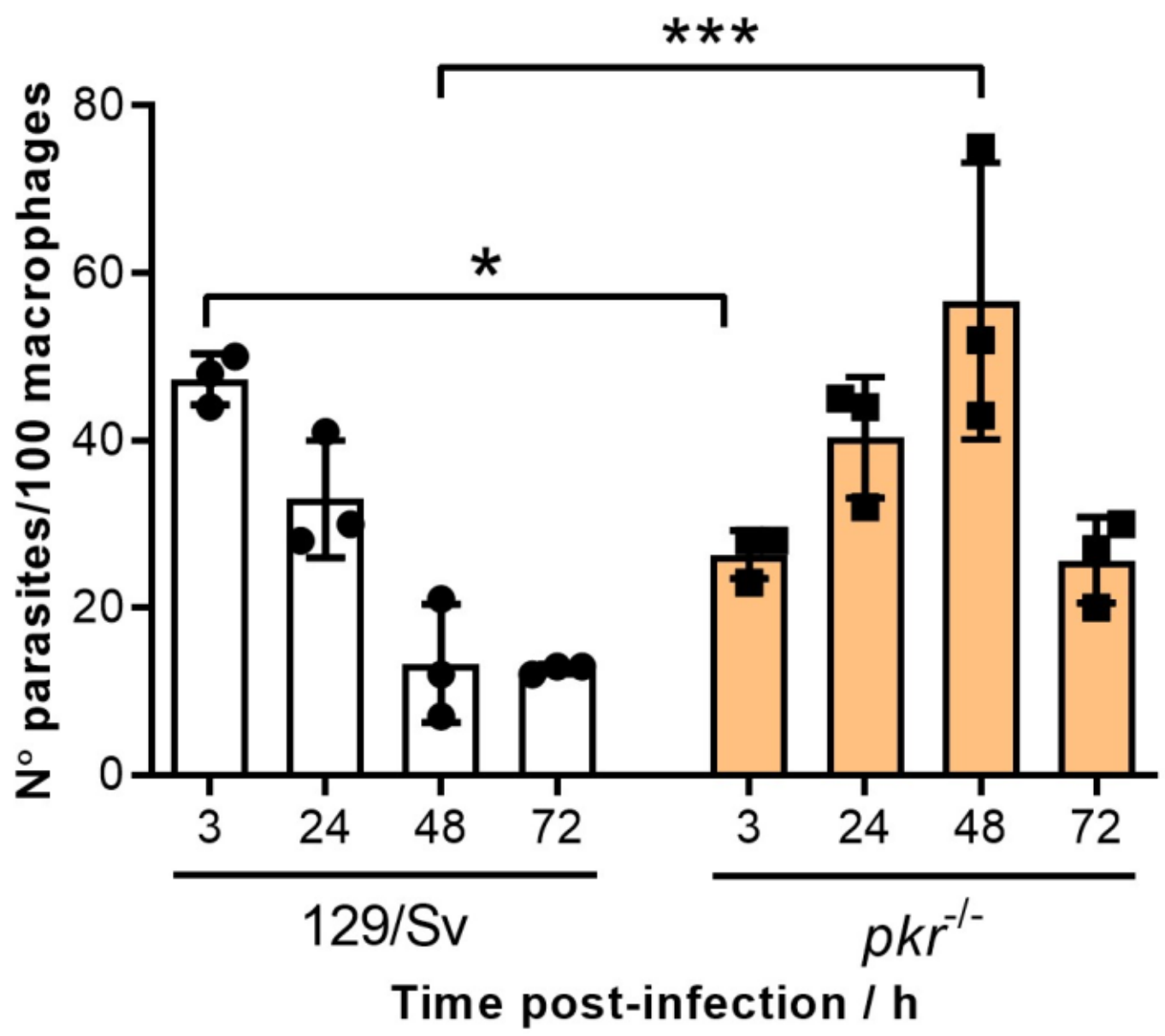


**Supplementary Fig. 3**: **Protein kinase R modulates macrophage infection by *L. infantum*.** Peritoneal thioglycolate-recruited macrophages from 129/Sv and protein kinase R (PKR)-knockout mice (*pkr*$^{-/-}$) cultivated on glass coverslips were infected with promastigotes at a 5:1 parasite:macrophage ratio in RPMI-BSA at 37°C. After 3 h, the monolayers were washed to remove extracellular parasites, fixed with methanol and Giemsa-stained, or further incubated for the indicated times in RPMI-FCS at 37°C before fixation and staining. The number of intracellular parasites was determined under the light microscope. N=1, with three biological replicates. Statistical significance was assessed using two-way ANOVA with **(A)** only Sidak's multiple comparisons tests. Asterisks over bars indicate significant differences compared to the data for all other timepoints within the group; *P <0.05, ***P <0.001.

Supplementary Figure 4

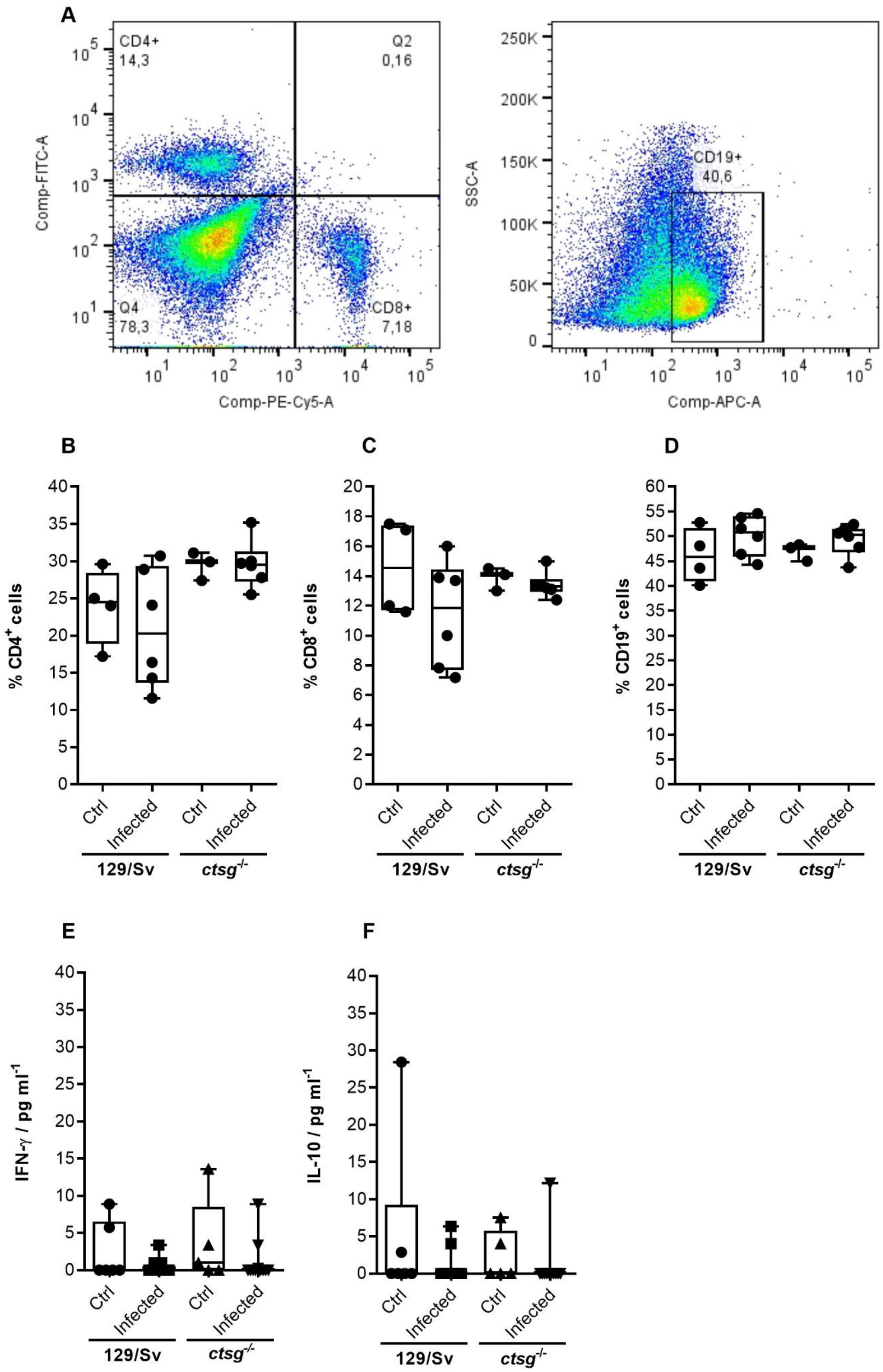

**Supplementary Fig. 4 Gating strategy for identification of lymphocyte subpopulations.** 129/Sv and cathepsin G-knockout (*ctsg*$^{-/-}$) mice were infected by retro-orbital inoculation of *L. infantum* promastigotes ($10^7$ parasites/animal in PBS). Uninfected mice were used as a control (Ctrl). After 1 week, mice were euthanized and the spleens were removed and homogenized. The homogenates were centrifuged and the supernatants were collected for determination of cytokine levels by ELISAs. Cell suspensions were then counted to give the total cells per spleen and then stained with antibodies to determine lymphocyte subpopulations by flow cytometry. Cells were first gated to exclude debris using the FSC vs. SSC plot followed by FSC-H vs. FSC-A to exclude doublets (as in Supplementary Fig. 5). $CD4^+$ and $CD8^+$ cells were gated on a CD4 vs. CD8a plot, while $CD19^+$ cells were identified on an individual plot against SSC **(A)**. Graphs show the percentage of **(B)** $CD4^+$, **(C)** $CD8^+$, and **(D)** $CD19^+$ cells, and ELISA of splenocytes for **(E)** IFNγ and **(F)** IL10. Each point represents an individual mouse and graphs are shown as box and whisker plots with the median (center line), lower and upper quartiles ($25^{th}$ and $75^{th}$ percentiles; box limits), and min/max (whiskers). Statistical analysis was performed using one-way ANOVA with Bonferroni posttest. *** $P < 0.001$.

Supplementary Figure 5

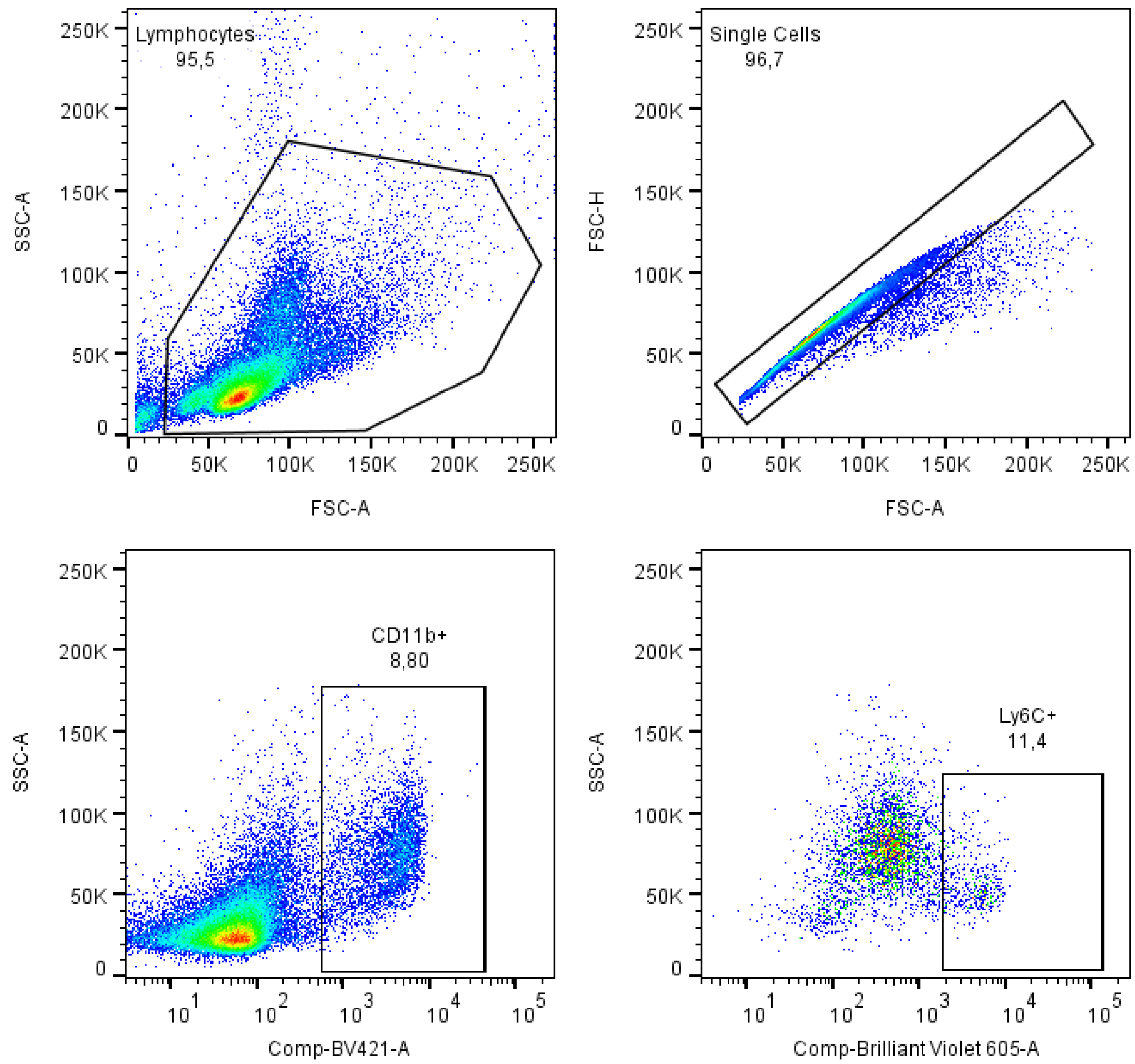


**Supplementary Fig. 5 Gating strategy for identification of leukocyte subpopulations.** 129/Sv and cathepsin G-knockout (*ctsg*$^{-/-}$) mice were infected by retro-orbital inoculation of *L. infantum* promastigotes ($10^7$ parasites/animal in PBS). Uninfected mice were used as a control (Ctrl). After 1 week, mice were euthanized and the spleens were removed and homogenized. The homogenates were centrifuged and the supernatants were collected for determination of cytokine levels by ELISAs. Cell suspensions were then counted then stained with antibodies to determine leukocyte subpopulations by flow cytometry. Cells were first gated to exclude debris using the FSC vs. SSC plot **(A)**, followed by FSC-H vs. FSC-A to exclude doublets **(B)**. CD11b$^+$ cells were selected from a SSC vs. CD11b plot **(C)**. From the CD11b$^+$ cells, Ly6C$^{hi}$ cells were selected from a SSC vs. Ly6C plot **(D)**.
