# Supplementary material for: Cathepsin G and neutrophil elastase control Leishmania infantum parasitism through TLRs-dependent induction of IFN-α and TNF-α
Source: Commun Biol. 2026 Jun 29;9:1373. doi: 10.1038/s42003-026-10555-8 (PMC13683408; doi:10.1038/s42003-026-10555-8)
Supplement: Supplementary file 2 [file 42003_2026_10555_MOESM4_ESM.pdf]

nature portfolio

Corresponding author(s): Ana Paula C A Lima

Last updated by author(s): May 17, 2026

Reporting Summary

Nature Portfolio wishes to improve the reproducibility of the work that we publish. This form provides structure for consistency and transparency in reporting. For further information on Nature Portfolio policies, see our Editorial Policies and the Editorial Policy Checklist.

Statistics

For all statistical analyses, confirm that the following items are present in the figure legend, table legend, main text, or Methods section.

n/a	Confirmed	
☐	☒	The exact sample size (*n*) for each experimental group/condition, given as a discrete number and unit of measurement
☐	☒	A statement on whether measurements were taken from distinct samples or whether the same sample was measured repeatedly
☐	☒	The statistical test(s) used AND whether they are one- or two-sided<br>*Only common tests should be described solely by name; describe more complex techniques in the Methods section.*
☐	☒	A description of all covariates tested
☐	☒	A description of any assumptions or corrections, such as tests of normality and adjustment for multiple comparisons
☐	☒	A full description of the statistical parameters including central tendency (e.g. means) or other basic estimates (e.g. regression coefficient) AND variation (e.g. standard deviation) or associated estimates of uncertainty (e.g. confidence intervals)
☐	☒	For null hypothesis testing, the test statistic (e.g. *F*, *t*, *r*) with confidence intervals, effect sizes, degrees of freedom and *P* value noted<br>*Give P values as exact values whenever suitable.*
☒	☐	For Bayesian analysis, information on the choice of priors and Markov chain Monte Carlo settings
☒	☐	For hierarchical and complex designs, identification of the appropriate level for tests and full reporting of outcomes
☒	☐	Estimates of effect sizes (e.g. Cohen's *d*, Pearson's *r*), indicating how they were calculated

Our web collection on statistics for biologists contains articles on many of the points above.

Software and code

Policy information about availability of computer code

Data collection	n/a
Data analysis	n/a

For manuscripts utilizing custom algorithms or software that are central to the research but not yet described in published literature, software must be made available to editors and reviewers. We strongly encourage code deposition in a community repository (e.g. GitHub). See the Nature Portfolio guidelines for submitting code & software for further information.

Data

Policy information about availability of data

All manuscripts must include a data availability statement. This statement should provide the following information, where applicable:

- Accession codes, unique identifiers, or web links for publicly available datasets
- A description of any restrictions on data availability
- For clinical datasets or third party data, please ensure that the statement adheres to our policy

n/a

Research involving human participants, their data, or biological material

Policy information about studies with human participants or human data. See also policy information about sex, gender (identity/presentation), and sexual orientation and race, ethnicity and racism.

Reporting on sex and gender	n/a
Reporting on race, ethnicity, or other socially relevant groupings	n/a
Population characteristics	n/a
Recruitment	n/a
Ethics oversight	n/a

Note that full information on the approval of the study protocol must also be provided in the manuscript.

Field-specific reporting

Please select the one below that is the best fit for your research. If you are not sure, read the appropriate sections before making your selection.

☒ Life sciences ☐ Behavioural & social sciences ☐ Ecological, evolutionary & environmental sciences

For a reference copy of the document with all sections, see nature.com/documents/nr-reporting-summary-flat.pdf

Life sciences study design

All studies must disclose on these points even when the disclosure is negative.

Sample size	T n-sample calculation for two-tailed t-test for independent samples was performed on the website powerandsampesize.com (http://powerand samplesize.com/Calculators/Compare-2-means/2- Sample-Equality). We chose the size depending on the confidence interval according to CHOW; SHAW; WANG 2008, and the following parameters were applied: Stastitical power 90%; Statistical signifiance threshold p<0.05; Estimated difference between groups 20%; expected standard deviation 15%; Sampling ratio: 1 (considering comparison of groups with the same n).
Data exclusions	Data was analysed using the Outlier calculator on Prism at: https//www.graphpad.com/quickcalcs/grubbs1/. When outliers were found, they were excluded from the compiled dataset for the calculations of median or average, and standard deviations.
Replication	Experiments were perfomed at least 2 independent times (with more than one biological replicate each time) to confirm reproducibility. The number of replicates is described in figure legends, or in the excel supplementary sheet containing the raw data.
Randomization	Mice of approximate ages were randomly distributed in the experimental groups.
Blinding	To comply with safety rules of the animal house of UFRJ, cages containing infected animals had to be labelled specifying the mice and infection agent; so data collaection was not blinded.

Reporting for specific materials, systems and methods

We require information from authors about some types of materials, experimental systems and methods used in many studies. Here, indicate whether each material, system or method listed is relevant to your study. If you are not sure if a list item applies to your research, read the appropriate section before selecting a response.

Materials & experimental systems

n/a	Involved in the study	
☐	☒	Antibodies
☒	☐	Eukaryotic cell lines
☒	☐	Palaeontology and archaeology
☐	☒	Animals and other organisms
☒	☐	Clinical data
☒	☐	Dual use research of concern
☒	☐	Plants

Methods

n/a	Involved in the study	
☒	☐	ChIP-seq
☐	☒	Flow cytometry
☒	☐	MRI-based neuroimaging

Antibodies

Antibodies used

anti-Fc-γ III/II (CD16/32) receptor antibody (BioLegend) ; Fluorochrome-labs rat anti-mouse antibodies as follows: BV421-CD11b clone M1/70 (BioLegend, cat# 101236), BV605-Ly6C clone HK1.4 (BioLegend, cat # 128031), FITC-CD4 clone GK1.5 (eBioscience cat# 11-0041-85), PE-Cy5-CD8 clone 53- 6.7 (eBioscience cat # 15-0081-83), and APC-CD19 clone MB19-1(eBioscience cat # 17-0191-82); R&D Systems DuoSet® ELISA kits: TNF-α, IFN-γ, IL-10, IL-12p40, CCL2, CX3CL1, CXCL2 (also known as MIP-2), and CCL5 (also known as RANTES).

Validation

all antibodies were validated by the manufacturer

Animals and other research organisms

Policy information about studies involving animals; ARRIVE guidelines recommended for reporting animal research, and Sex and Gender in Research

Laboratory animals

Mus musculus male and female mice, aged between 8–14 weeks ; C57BL/6J (Central animal facility of UFRJ); Neutrophil elastase (NE)-knockout (ela2-/-) Kindly donated by Dr. Bernhard Ryffel, Centre National de la Recherche Scientifique (CNRS), Orleans, France (own colony maintained at the Laboratory of Transgenic Animals at UFRJ, LAT); Toll-like receptor 2 (TLR2)-knockout (tlr2-/-) Kindly donated from the colony of Prof. Marcelo T. Bozza (UFRJ); Toll-like receptor 3 (TLR3)-knockout (tlr3-/-) Kindly donated from the colony of Dr. Renata Meirelles Pereira (UFRJ); Protein kinase R (PKR)-knockout (pkr-/-) 129 S/v background Kindly donated by Dr. Aristóbolo M. Silva, Universidade Federal de Minas Gerais (UFMG), Minas Gerais, Brazil; Cathepsin G (CG)-knockout (ctsg-/-) from Cathepsin G (CG)-knockout (ctsg-/-) (Own colony maintained at LAT of UFRJ); IFN-α/β receptor alpha chain (IFNAR)-knockout (ifnar-/-) 129 S/v background Kindly donated from the colony of Dr. Herbert L. M. Guedes (UFRJ).

Wild animals

n/a

Reporting on sex

peritoneal macrophages from either male or female mice were collected for in vitro experiments and provided similar results; for in vivo experiments both male and female mice were used in each experiments and the the results from independent experiments were compiled ; it was not planned to perform sex-separated experiments; sex-bases differences were not analysed.

Field-collected samples

n/a

Ethics oversight

The protocols approved by the ethics committee of UFRJ (Comissão de Ética no Uso de Animais (CEUA)), under the license 173/19-UFRJ.

Note that full information on the approval of the study protocol must also be provided in the manuscript.

Plants

Seed stocks

n/a

Novel plant genotypes

n/a

Authentication

n/a

Flow Cytometry

Plots

Confirm that:

☒ The axis labels state the marker and fluorochrome used (e.g. CD4-FITC).

☒ The axis scales are clearly visible. Include numbers along axes only for bottom left plot of group (a 'group' is an analysis of identical markers).

☒ All plots are contour plots with outliers or pseudocolor plots.

☒ A numerical value for number of cells or percentage (with statistics) is provided.

Methodology

Sample preparation

mice were euthanized, and the spleens were removed and homogenised, cells were centifuged and ressupended in an ACK solution for 2 min, and then supplemented with complete medium. ACK-treated single-cell suspensions from the spleen (107 cells) were washed in PBS at 300×g for 5 min, ressuspened in PBS, quantified by counting cells in a Neubauer under the

microscope. Suspensions were incubated with an anti-Fc-γ III/II (CD16/32) receptor antibody (BioLegend) for 10 min, then stained with fluorochrome-conjugated antibodies for 30 min. Cell numbers in the suspensions were quantified, and then incubated with fluorochrome-labeled antibodies, before flow cytometry.

Instrument	FACSCanto™ II (BD Biosciences)
Software	FlowJo v10
Cell population abundance	populations were not sorted (n/a)
Gating strategy	To identify leukocyte sub-populations: Cells were first gated to exclude debris using the FSC vs. SSC plot, followed by FSC-H vs. FSC-A to exclude doublets; CD11b+ cells were selected from a SSC vs. CD11b plot, From the CD11b+ cells, Ly6Chi cells were selected from a SSC vs. Ly6C plot.). To identfy lymphocytes: Cells were first gated to exclude debris using the FSC vs. SSC plot followed by FSC-H vs. FSC-A to exclude doublets, CD4+ and CD8+ cells were gated on a CD4 vs. CD8a plot, while CD19+ cells were identified on an individual plot against SSC .

☒ Tick this box to confirm that a figure exemplifying the gating strategy is provided in the Supplementary Information.
