# Supplementary material for: Cathepsin G and neutrophil elastase control Leishmania infantum parasitism through TLRs-dependent induction of IFN-α and TNF-α
Source: Commun Biol. 2026 Jun 29;9:1373. doi: 10.1038/s42003-026-10555-8 (PMC13683408; doi:10.1038/s42003-026-10555-8)
Supplement: Supplementary file 3 [file 42003_2026_10555_MOESM2_ESM.docx]

**Description of Additional Supplementary Files**

**File name:** Supplementary Data

**Description:** Raw data used in figures.
